# Is population structure in the European white stork determined by flyway permeability rather than translocation history?

**DOI:** 10.1002/ece3.845

**Published:** 2013-11-07

**Authors:** Jill M Shephard, Rob Ogden, Piotr Tryjanowski, Ola Olsson, Peter Galbusera

**Affiliations:** 1 Centre for Research and Conservation - Royal Zoological Society of AntwerpKoningen Astridplein 26, 2018, Antwerp, Belgium; 2 School of Veterinary and Life Sciences, Murdoch UniversityMurdoch, Western Australia, 6150, Australia; 3 Royal Zoological Society of Scotland, Edinburgh Zoo134 Corstorphine Road, Edinburgh, EH12 6TS, UK; 4 Institute of Zoology, Poznan University of Life SciencesWojska Polskiego 71 C, 60-625, Poznań, Poland; 5 Department of Ecology, Animal Ecology, Lund UniversitySE-223 62, Lund, Sweden

**Keywords:** Admixture, *Ciconia ciconia*, population decline, population genetics, reintroduction

## Abstract

European white stork are long considered to diverge to eastern and western migration pools as a result of independent overwintering flyways. In relatively recent times, the western and northern distribution has been subject to dramatic population declines and country-specific extirpations. A number of independent reintroduction programs were started in the mid 1950s to bring storks back to historical ranges. Founder individuals were sourced opportunistically from the Eastern and Western European distributions and Algeria, leading to significant artificial mixing between eastern and western flyways. Here we use mitochondrial and microsatellite DNA to test the contention that prior to translocation, eastern and western flyways were genetically distinct. The data show a surprising lack of structure at any spatial or temporal scale suggesting that even though birds were moved between flyways, there is evidence of natural mixing prior to the onset of translocation activities. Overall a high retention of genetic diversity, high *N*_*ef*_, and an apparent absence of recent genetic bottleneck associated with early 20th century declines suggest that the species is well equipped to respond to future environmental pressures.

## Introduction

Long-distance cyclic migrants can display patterns of genetic structure or panmixia dependent on behavioral and/or environmental drivers. In particular, the relationship between breeding and overwintering sites can significantly influence the genetic structuring of populations. For example, among broadly distributed species that diverge to geographically separate overwintering grounds, nest site philopatry, monogamy, and the degree to which migratory pathways are behaviorally entrenched will all serve to impact upon genetic structure. A lack of mixing between divergent migration groups during overwintering periods is inconsequential in terms of gene flow. However, the degree of admixture between groups on returning to the summer breeding grounds both within and between populations will likely determine genetic structure. The level of adherence to migratory pathways over consecutive years will add to this effect, as will natal dispersal distance.

Sutherland (1998) suggests that current Northern European migration systems may only have developed in the last ten thousand years since the last Ice Age, and may be subject to significant levels of flexibility within species. For instance, some bird species are shown to exhibit “flyway permeability,” in which birds switch between flyways in response to social cues, or environmental pressure (Guillemain et al. 2005). Typically, species with geographically divergent migratory pathways and destinations are genetically distinct (Guillemain et al. 2005; Irwin et al. 2011), while those with divergent overwintering grounds but similar breeding grounds display a lack of genetic divergence (Dallimer et al. 2003; Davis et al. 2006).

**Figure 1:**
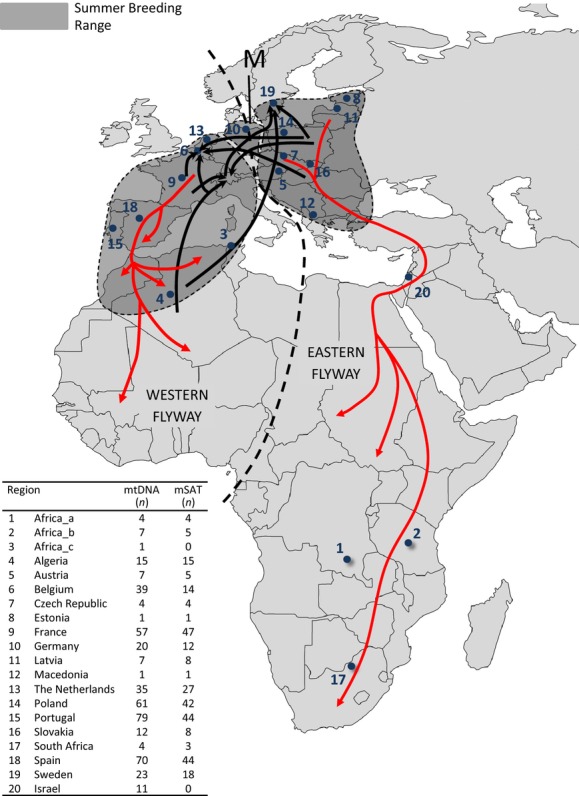
Distribution of sample regions (*n* = 20) within the eastern and western flyway. The black dotted line shows the suggested flyway division. Approximate summer breeding ranges are indicated with dark gray shading. The central breeding range (M) indicates where birds are known to choose either flyway. Red arrows indicate the direction of overwintering migration in each flyway. The black arrows show the translocation routes of individuals between flyways as part of reintroduction activities. The inset table shows the name of each region and the sample sizes for both the mitochondrial (*n* = 458 + 1 Reference Sequence of unknown origin) and microsatellite data (*n* = 295).

The European white stork (*Ciconia ciconia*) has an extensive European breeding distribution covering most of continental Europe as far east as Belarus and Ukraine and as far north as southern Scandinavia (Fig. 1). South of the Mediterranean they breed in northern Algeria and Morocco. The European population is long considered to diverge to eastern and western migration pools as a result of independent overwintering flyways, which have also been considered as genetically divergent. This notional divide occurring in the vicinity of the Elbe River in Germany is based on observed migration behavior, with western individuals migrating across the Straits of Gibraltar to overwintering areas in the Sahel region in West Africa and eastern birds following a path through Istanbul to overwintering areas in East and South Africa (Fig. 1; Hagemeijer and Blair 1997). However, notably some birds close to this divide are known to migrate along either flyway (NABU 2013).

Since the 1930s, a small but consolidated breeding population has established near Cape Town, South Africa, at the southerly extent of the eastern population migratory path (Harrison et al. 1997). Data from dispersal studies are limited, but in Poland, median dispersal distances are only 26 km, although have been recorded at 462 km from the natal site (Chernetsov et al. 2006). While Chernetsov et al. (2006) failed to find that dispersal was density dependent, females were found to settle further from the natal site than males. Alternatively, Itonaga et al. (2011) found both age and density dependence were important determinants of dispersal in at least one East German population due potentially to increased competition from recent population growth. There are no similar studies for the western distribution. However, in mature birds, satellite tracking evidence over consecutive seasons suggests that individual birds, although highly vagile, do show significant nest site philopatry and follow remarkably similar migration routes over many years (Berthold et al. 2004; Rycken 2011). It is unclear to what extent this is controlled by inherited genetic information (*sensu* Mueller et al. 2011). Manipulation experiments demonstrate that social training may play a large part in the cultural transmission of migration flyways (Chernetsov et al. 2004). Similarly, recent satellite tracking reveals consistent changes to migration behavior in some western populations with birds overwintering in Spain rather than Africa (Rycken 2011). However, in European white stork, neither migration direction nor distance will determine genetic structure. For example, a reproductively mature bird from the east that winters in Spain, but returns to the east to breed will not impact genetic structure. Rather movement in and out (natal dispersal) of breeding grounds with subsequent breeding activity will determine the extent of “flyway permeability”.

In addition, current patterns of population structure are complicated by reintroduction activities that began in the mid 1900s. Significant demographic changes are recorded since the 1930s, with country-specific extirpations throughout the western distribution and Sweden since the late 19th century, although a declining stronghold population remained in the Iberian Peninsula during this time (Bairlein 1991). Decline has been linked to habitat alteration including wetland loss and agricultural intensification and is also associated with hunting, overhead powerlines, drought in the wintering areas, and heavy rain during the breeding season (Saether et al. 2006; Thomsen and Hötker 2006). Natural population re-establishment in the western range and Sweden by means of natal or breeding dispersal was seen as unlikely, and starting in 1948, independent captive breeding and reintroduction programs in Switzerland, France, Germany, Belgium, the Netherlands and Sweden returned natural breeding populations to historical ranges. During the same period, eastern populations showed some regional population fluctuations and range extensions, but on the whole remained relatively stable or showed slight increases (Bairlein 1991). Choice of reintroduction stock was arbitrary and linked to availability and logistical considerations rather than empirical information (Bloesch 1980). In the Swiss program, the original founders were obtained from resident Algerian populations considered part of the western distribution (Bloesch 1980; Schaub et al. 2004). Stock from these birds was used in the Swedish program, with numbers subsequently bolstered by additional birds from Northeastern Europe (Olsson 2007). Native Swedish storks were considered to belong to the Eastern European population (Schulz 1998). Similarly, founder individuals in the Belgian program were opportunistically sourced from the western and eastern populations (Struyf 1991).

As a result, significant artificial mixing occurred between the two flyways (Fig. 1). In response to this, concerns have been raised about the geographic origin of translocated storks, and the effect that translocation may have had on patterns of population genetic structure, and therefore individual biology. In particular, reproductive failures have been reported in Swedish founder birds sourced from North African stock that are proposed to be due to outbreeding depression (Olsson 2007).

Here we test the contention that prior to translocation, eastern and western migration flyways were genetically distinct. Further, we test that as a consequence of reintroduction activities, the artificial mixing of putatively genetically distinct migration pools has led to the unintended homogenization of contemporary populations. As current populations of European white stork probably contain translocated captive-bred ancestors, we have split our sampling to historical and contemporary sets. The historical sample (prior to the onset of reintroduction activities in the 1950s) is used as a reference to identify natural levels of admixture as opposed to admixture as a consequence of translocation. Finally, given recent heavy population declines in parts of the species range, we test for evidence of genetic bottleneck.

## Methods

### Sampling and DNA extraction

Samples (blood, feather, dried skin, tissue in EtOH) were collected from throughout the range of the European White Stork from both wild (*n* = 411) and captive birds of known origin (*n* = 47) and were allocated to 20 geographic regions (Fig. 1). Of these, 54 samples were obtained from museum collections. Museum tissue (dried toe pad) was excised using fresh gloves and a sterile scalpel blade for each specimen. Museum sample information and accession codes are given in Table S1. Most contemporary samples were collected during standard ringing sessions during the breeding season. South African samples were collected from a newly established resident population. Samples from Israel were collected from resident birds, but also opportunistically from birds actively migrating or injured who could be resident elsewhere, and were presumed to belong to the eastern flyway. Birds sampled within Belgium and Sweden belong to semisupported free-flying colonies with natural potential for recruitment and dispersal. The Belgian samples were sourced from Planckendael Wildlife Park, which functioned as a closed reintroduction population in the 1970–1980s (Struyf 1991), and received founder stock from the eastern and western flyway (Fig. 1). The Swedish population also contains descendants of another reintroduction site, which contains birds of 100% Algerian origin (western flyway; sourced through the Swiss reintroduction program from the 1950s), hybrid individuals from Algerian and Northern European stock, and wild birds of 100% Northern European ancestry. In both instances, the North European stock was associated with the eastern flyway. Samples sourced from within colonies were only used if unrelated (based on pedigree data). All other samples were considered unrelated. Logically all samples from the western flyway or Swedish sites taken after the 1950s could represent or contain descendants of reintroduction stock and therefore could contain genetic profiles from either flyway.

For analysis purposes, sample regions were aggregated to west (western flyway) or east (eastern flyway) migration pools according to Fig. 1. Satellite tracking evidence suggested that birds sourced from Germany and Austria may use both migration flyways and were allocated to an intermediate class (M). Where appropriate, they were excluded from some analyses.

Samples were also divided temporally to “historical” or “contemporary” groups. Historical samples were defined as those sourced prior to reintroduction activities. These samples were obtained from museum collections spanning the years 1829 to 1954 (Av. 1910). As mentioned previously, the historical sample set was used as a reference to identify natural levels of admixture as opposed to admixture as a consequence of translocation during reintroduction activities.

Genomic DNA was extracted from feather, tissue, blood, and museum skins, using the Puregene® DNA Purification Kit (Gentra®). Protocols varied per source type, but followed closely the manufacturer's instructions for solid tissue and compromised blood. Museum tissue was rehydrated for 24–48 h in 9% saline solution prior to extraction. All extractions were performed in a laminar flow unit with dedicated stocks for both contemporary and museum materials. Extractions and PCR preparation were performed in separate purpose-built rooms including a custom PCR hood, using dedicated equipment, filtered tips, and appropriate negative controls.

### Mitochondrial methods and analysis

A 373-bp fragment of mitochondrial control region (Domain 1) was amplified using the primers CR57F (5′GGG AAA TGT ACT AGC TGA CTG3′) and CR465R (5′ CCT GTA CAG ACC CAA ACC ATA G3′), or the following internal primer combinations: CR57F and CR214R (5′ATC CAC GCA TCA TTT CAA CA3′); CR195F (5′TGT TGA AAT GAT GCG TGG AT3′) and CR334R(5′CTA TCC CCT TGG GAG ACC TG3′); CR315F (5′ACC TAG GGG AGG ACT GGA GA3′) and 465R for difficult and museum samples. *C. ciconia* displays the standard avian gene order. The complete mtDNA is available on GenBank (NC_002197) and was used as a reference sequence. Successful sequence was obtained from all museum samples using the three overlapping internal primer pairs with forward and reverse sequencing where required. These primers amplified short fragment reads between 130 and 160 bp in length to cater for low-copy or degraded source material. The three overlapping sequences were appended to make the complete 373-bp sequence. In most cases, multiple sequence reads were obtained for museum samples. Only those data providing unequivocal sequence and peak reads were used. Primer names match the base pair number of the reference sequence. A subset of individuals was re-extracted using an alkaline lysis protocol (Tamura and Aotsuka 1988) to rule out the occurrence of a nuclear pseudogene.

PCR mixture contains 1 μL 10 × buffer, 0.4 μL of 1 mmol/L MgCl_2_, 0.2 μL 10 mmol/L dNTP's, 0.2 μL of each primer (10 μmol/L per μL), 0.05 μL of AmpliTaq Gold DNA Polymerase (Applied Biosystems, Foster City, CA), and 30–100 ng of DNA template adjusted to a final volume of 10 μL with ddH_2_O. Reactions from museum tissues included 0.4 μL of BSA (0.8 μg/μL). PCR cycles were as follows: denaturation for 1 min 30 s at 95°C followed by 50 cycles of 94°C for 45 s, 55°C to 60°C for 45 s, 72°C for 45 s, with a final extension of 72°C for 10 min. All products were visualized on a 1.6% TAE agarose gel along with negative extraction and PCR controls. Sequences were run on an ABI 3730xl DNA analyzer.

Sequences were aligned and checked in BIOEDIT (Hall 1999) against a reference sequence (GenBank Accession No: NC_002197). Unique haplotypes were identified using DnaSP version 5.00.07 (Librado and Rozas 2009). Haplotype and nucleotide diversities were calculated in Arlequin version 3.5 (Excoffier and Lischer 2010) at different spatial (region and migration pool) and temporal scales (historical and contemporary) as described above and based on the nucleotide substitution model of Tamura and Nei with a gamma value of 0.481 and the corrected Akaike information criterion for small sample size (AICc). This was the closest model to the one inferred (TIM1+I+G) by jModelTest version 0.1.1 (Posada 2008).

The evolutionary relationship of haplotypes was calculated in TCS (Clement et al. 2000) with the resulting network summarized according to migration and temporal pools. Network topology and haplotypes with the greatest out-group probability were identified by the algorithm with 95% probability. Demographic bottleneck or expansion was inferred using Tajima's *D* (Tajima 1989) and Fu's *F* (Fu 1997), respectively. Mismatch variance was calculated at each level in the data. Mismatch plots were generated for those data showing a significant Fu's *F*. Time since expansion and effective female population size were calculated based on the formula's *τ* = 2*ut* and *N*_ef_ = *θ*_1_/2*u*, respectively (Rogers and Harpending 1992), where *τ* = units of mutational time, *u* = the mutation rate over the fragment assayed (i.e., *u* = mr × 10^−8^ × fragment length), and *θ*_1_ is a correlate of final population size (Rogers and Harpending 1992; Excoffier and Lischer 2010). The mismatch distribution between observed and expected differences was compared with that expected under the sudden expansion model of Rogers and Harpending (1992) and was calculated using the least squares method of Schneider and Excoffier (1999). It was tested for significance using a coalescent approach adapted from Hudson (1990) and an average rate of 20% divergence per million years (Domain 1 of the CR in birds; Baker and Marshall 1997). All demographic calculations were performed in Arlequin based on 10000 replications. A lack of population structure and a number of other assumption violations precluded the use of other geneology samplers such as Migrate-n (Beerli and Palczewski 2010), Lamarc (Kuhner 2006), or IMa2 (Hey 2010).

To avoid the constraint of imposing a group structure on the data, we used a bayesian spatial clustering approach implemented in BAPS version 5.3 (Corander and Tang 2007; Corander et al. 2008), using individual sequence data and the admixture model. Admixture analysis was performed with and without spatial information based on the mixture clustering output following the recommendations of Corander et al. (2009) at each temporal scale.

Spatial analysis of shared alleles (SAShA; Kelly et al. 2010) was used to identify which haplotypes may be important in driving the spatial association of haplotypes. This analysis uses spatial and allele (haplotype) information to detect nonrandom allele distribution against an expectation of panmixia. The test statistic OM describes the observed mean distance between alleles. Where OM is less than the expected mean (EM), alleles are considered to be aggregated (underdistributed). Where OM is larger than EM, alleles are considered overdispersed (panmictic). A jackknifing procedure allows the identification of which alleles contribute to the distribution.

### Microsatellite genotyping and analysis

Two hundred and ninety-five samples were genotyped for nine loci previously reported in Shephard et al. (2009); Fig. 1). Thirty-two individuals represented the historical set and 263 the contemporary sample. Loci ws*μ*03, Cc06, Cc07, ws*μ*20, and ws*μ*23 products were amplified in singleplex in the following 10 μL PCRs containing 0.25 units ABgene Thermo-Start DNA Polymerase, 1× Thermo-Start® reaction buffer, 2.5 mmol/L MgCl_2_, 0.2 mmol/L each dNTP, 20 pmol primer, and 1 μL of template DNA. For loci ws*μ*14, ws*μ*17, Cc01, and Cc03, a multiplex PCR was performed using Qiagen Type-it® Multiplex PCR kit with 0.17 pM of each primer and 1 μL of template DNA. Reaction conditions for all were as follows: 95°C (15 min for those using ABgene Thermo-start DNA polymerase or 5 min for Qiagen Type-it®); 5 cycles of 95°C for 30 s, 54°C for 1 min 30 s, 72°C for 30 s; 30 cycles of 95°C for 30 s, 52°C for 1 min 30 s, 72°C for 30 s; followed by 60°C for 30 min. Fragment size was resolved on an ABI 3730xl genetic analyzer relative to the LIZ500 size standard (Applied Biosystems) and analyzed using GeneMapper version 4 (Applied Biosystems). We tested for deviations from Hardy–Weinberg and linkage disequilibrium in GENEPOP 4.0 (Rousset 2008) using both Bonferroni and B-Y FDR methods (Rice 1989; Benjamini and Yekutieli 2001; Narum 2006), and the presence of null alleles using MICRO-CHECKER (Van Oosterhout et al. 2004). Observed and expected heterozygosities and the mean number of alleles were calculated in GENETIX version 4.05.02 (Belkhir et al. 1996–2004). To take into account differences in sample size, allelic richness was estimated for samples belonging to different regions (east versus west) or periods (historical versus contemporary) in FSTAT version 1.2 (Goudet 1995).

To infer levels of population genetic differentiation between regions, we calculated D_*est*_ across all loci with SMOGD version 1.2.5 (Crawford 2010) and F_*ST*_ in GENEPOP using parameter *θ*. The hypothesis of east versus west differentiation was further explicitly tested using AMOVA in ARLEQUIN.

The likelihood of bottleneck/expansion was assessed in the east and the west pool using BOTTLENECK version 1.2.02 (Piry et al. 1999) where significant heterozygote excess or deficiency indicates bottleneck or expansion, respectively. Data were analyzed under three different mutation models: infinite allele model (IAM), stepwise mutation model (SMM), and two-phase model (TPM). For the latter, combinations of 95% single-step mutations and 5% multistep mutations were used, with a variance of 30 among multiple-step mutations (10000 replications; Piry et al. 1999). Given the sample sizes, this test was only performed for the contemporary sample set. The assumption of mutation/drift equilibrium in the estimation of D_*est*_ and F_*ST*_ was violated. Accordingly, we used STRUCTURE version 2.2 (nonspatial; Pritchard et al. 2000) and BAPS version 5.3 (spatial; Corander et al. 2009) to explore genetic structure and a factor correspondence analysis in GENETIX to visualize the genetic relationship between individuals. In STRUCTURE, each *k (*1–6) was run 3 times, with 10^6^ permutations and a burn-in of 10^5^ for each run, and an admixture model with correlated allele frequencies. As in the mtDNA analysis, STRUCTURE and BAPS were run at each temporal scale. Clustering was performed by individual within BAPS. The maximum number of clusters was set from 5 to 25 (with an interval of 5), and each maximum *k* value was repeated 5 times.

## Results

### Mitochondrial DNA

A total of 459 (including reference sequence) sequences were obtained of which 53 were from the historical set. One hundred and six haplotypes were identified (Tables S2 and S3; GenBank Accession Nos: JN410952-JN411056). The most common haplotype (CC003; Table S3) was found in 27% of the complete sample and in both migration pools. Notably, it was absent from Africa_a, Africa_b, and Africa_c, but not Algeria or South Africa. The five most common haplotypes described over 50% of the sample; 22 haplotypes were found in the historical data set, only two of which (CC011 and CC016) occurred in the contemporary sample. All other haplotypes occurred at an average frequency of 1.9%, and more than 50% of all haplotypes detected were singletons (Table S3). Haplotype diversity was very high (0.906 ± 0.0102), with a moderate level of overall nucleotide diversity (0.033 ± 0.0106; Table 1). There was little difference in either of these statistics when comparing between historical and contemporary data sets, or between migration pools. There were no noticeable differences in diversity levels among regions that had been involved in reintroduction projects with those that had not.

**Table 1:** Mitochondrial diversity statistics and neutrality tests by region, migration pool, and for the complete distribution. Belgium, the Netherlands, and Sweden contributed samples to the historical and contemporary analysis and were analyzed at each level. Regions with only one sample were excluded from “All Regions,” but included within “Migration Pool”. Data were analyzed in subsets; footnotes list those samples included in each level of the analysis. Values after haplotype and nucleotide diversity are standard errors. Numbers in brackets show significance levels where P(*D* sim < *D* obs) and P(sim *Fs* ≤ obs *Fs*). Values in bold are significant at *P* ≪ 0.05

Region	*n*	No. Haps	Hap Div	Nucleotide Div	Tajima's D (*D)*	Fu's F (*Fs*)	Mismatch Variance
All Regions							
East Africa (Africa_a and Africa_b)^1^	11	8	0.927 ± 0.067	0.01 ± 0.006	0.565 (0.31)	−2.44 (0.07)	3.991
Algeria	15	3	0.591 ± 0.077	0.0025 ± 0.002	−0.823 (0.24)	0.736 (0.63)	1.233
Austria	7	5	0.857 ± 0.137	0.0089 ± 0.006	−0.197 (0.46)	−0.612 (0.29)	4.929
Belgium	39	17	0.891 ± 0.029	0.0103 ± 0.006	−0.500 (0.36)	−**4.968 (0.02)**	4.341
Belgium – historical	7	7	1.000 ± 0.076	0.0076 ± 0.005	−0.963 (0.23)	−**4.774 (0.001)**	1.733
Belgium – contemporary	32	10	0.837 ± 0.036	0.0086 ± 0.005	0.357 (0.67)	−0.494 (0.44)	3.160
Czech Republic	4	2	0.500 ± 0.265	0.0014 ± 0.002	−0.612 (0.38)	0.172 (0.35)	0.300
France	57	31	0.941 ± 0.201	0.053 ± 0.026	0.324 (0.70)	−2.760 (0.23)	328.503
Germany	20	9	0.821 ± 0.073	0.024 ± 0.013	−**2.068 (0.006)**	1.528 (0.77)	227.910
Latvia	7	5	0.857 ± 0.1371	0.053 ± 0.034	−**1.457 (0.05)**	2.364 (0.84)	383.690
The Netherlands	35	8	0.642 ± 0.08	0.004 ± 0.002	−0.865 (0.21)	−1.889 (0.13)	3.177
The Netherlands – historical	17	4	0.418 ± 0.14	0.024 ± 0.001	−**1.825 (0.01)**	−0.661 (0.25)	1.785
The Netherlands – contemporary	18	5	0.719 ± 0.07	0.005 ± 0.004	−0.244 (0.45)	0.363 (0.58)	3.257
Poland	61	18	0.816 ± 0.046	0.017 ± 0.009	−**1.927 (0.005)**	−1.796 (0.28)	169.914
Portugal	79	32	0.958 ± 0.01	0.059 ± 0.029	0.685 (0.82)	−1.245 (0.43)	347.832
Slovakia	12	9	0.940 ± 0.058	0.040 ± 0.022	−**1.646 (0.04)**	−0.114 (0.45)	244.633
South Africa	4	3	0.50 ± 0.265	0.001 ± 0.002	−0.612 (0.37)	0.172 (0.32)	0.400
Spain	70	20	0.815 ± 0.041	0.030 ± 0.015	−0.870 (0.20)	−0.248 (0.53)	194.076
Sweden	23	13	0.889 ± 0.044	0.009 ± 0.005	−0.618 (0.30)	−**4.171 (0.03)**	5.090
Sweden – historical	11	6	0.727 ± 0.144	0.006 ± 0.004	−1.107 (0.14)	−1.558 (0.09)	3.926
Sweden – contemporary	12	7	0.803 ± 0.096	0.009 ± 0.005	−0.199 (0.45)	−0.171 (0.46)	5.361
Israel	11	7	0.873 ± 0.089	0.083 ± 0.044	0.986 (0.88)	3.635 (0.93)	na
Migration Pool							
Historical^2^							
East	27	15	0.883 ± 0.053	0.008 ± 0.005	−1.155 (0.13)	−**8.128 (<0.0001)**	3.658
West	28	12	0.714 ± 0.093	0.006 ± 0.004	−**1.684 (0.03)**	−**5.093 (0.005)**	5.508
Contemporary^3^							
East	98	27	0.819 ± 0.037	0.02 ± 0.011	−**1.583 (0.03)**	−2.915 (0.20)	201.658
West	270	61	0.911 ± 0.0122	0.039 ± 0.02	−0.209 (0.50)	−9.497 (0.07)	254.218
Total sample							
East	125	41	0.876 ± 0.0242	0.018 ± 0.01	−**1.660 (0.02)**	−**14.534 (0.002)**	159.636
West	298	70	0.921 ± 0.0101	0.037 ± 0.018	−0.487 (0.36)	−**17.136 (0.01)**	235.659
Complete Distribution	459	106	0.906 ± 0.0102	0.033 ± 0.016	−0.736 (0.27)	−**23.989 (0.003)**	208.503

1 This sample represents the historical population. There were no contemporary samples available for this region.

2 Regions represented are as follows: *East* – East Africa *n* = 11 (Africa_a and Africa_b), Estonia *n* = 1, Germany *n* = 1, Macedonia *n* = 1, Norway *n* = 1, Poland *n* = 1, South Africa *n* = 1, Sweden *n* = 11; *West* – Africa_c *n* = 1, Belgium *n* = 7, France *n* = 1, the Netherlands *n* = 17.

3 Samples from Austria and Germany not included as they may belong to either migration pool. Regions represented are as follows: *East* – Czech Rep *n* = 4, Latvia *n* = 7, Poland *n* = 60, Slovakia *n* = 12, South Africa *n* = 3, Sweden *n* = 12; *West* – Algeria *n* = 15, Belgium *n* = 32, France *n* = 56, the Netherlands *n* = 18, Portugal *n* = 79, and Spain *n* = 70.

**Figure 2:**
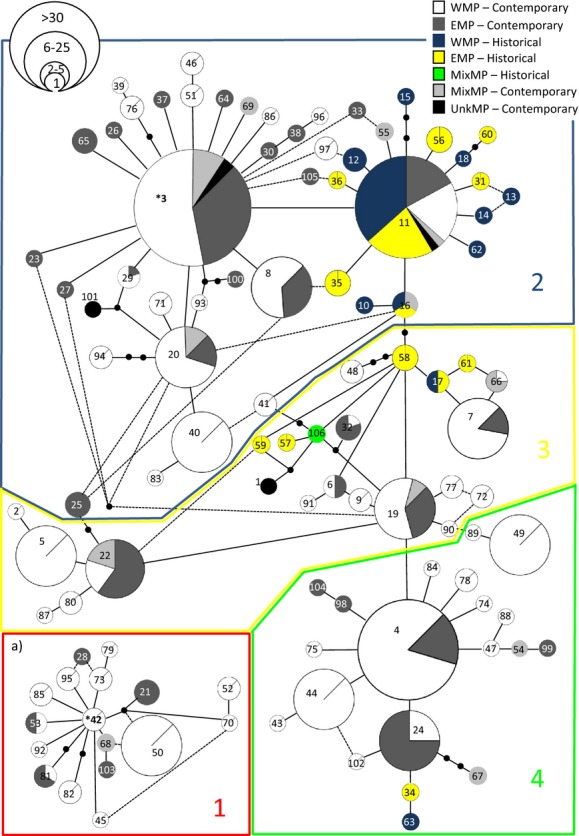
Network showing the evolutionary relationship between mtDNA haplotypes. Numbers correspond to Tables S2 and S3. Colored haplotypes show those in the historical sample. The proportion of haplotypes in each migration pool are shown in the pies where WMP = western migration pool; EMP= eastern migration pool; MixMP = individuals that may migrate either east or west; and UnkMP = unknown migration pool. Pie size indicates the proportion of individuals displaying each haplotype. Lines indicate a single base pair change between individuals. Solid small black circles indicate unsampled haplotypes. Dotted lines indicate unresolved connections between haplotypes. Inset a) indicates a second network that could not be joined to the main network. Haplotypes CC003 and CC042 (marked with an *) were designated as having the greatest out-group probability by the software. Colored boxes numbered 1–4 show which haplotypes occurred in each of the genetic clusters defined in the nonspatial admixture analysis in BAPS (Fig. 4a). The color of the box matches the color of the cluster in the BAPS plot (Fig. 4a).

Five regions showed signatures of population bottleneck (Table 1; Tajima's *D*), and these were predominantly from the eastern migration pool and the contemporary sample. The western migration pool showed a positive signature of bottleneck in the historical sample from the Netherlands. There were significant signatures of demographic expansion in Belgium and Sweden, for both east and west migration pools and for the distribution as a whole, which appears driven by historical processes (Table 1; Fu's *F*). Two distinct networks were identified by TCS (Fig. 2). The first shows a series of star-shaped structures. Haplotype 3 (CC003) was identified with the biggest out-group probability (0.08) by the software. The majority of haplotypes are clearly distributed among both migration pools. Colored haplotypes in Fig. 2 suggest some sort of clustering among historical samples. A second network contained 17 haplotypes. Haplotype CC042, which occurs in France, Portugal and Spain, was identified with the highest out-group probability (0.197; Fig. 2 – inset a).

**Figure 3:**
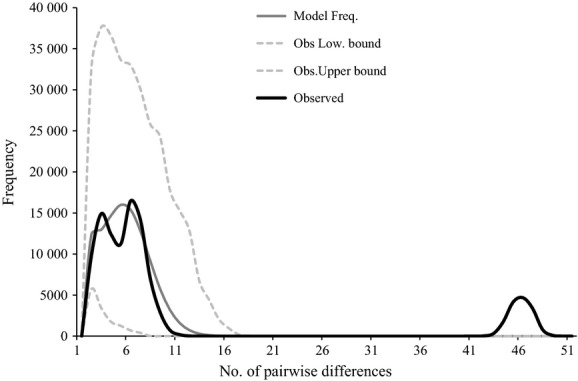
Mitochondrial DNA mismatch distribution showing the observed and expected distribution of pairwise differences used to test for deviation from the sudden expansion model (Rogers and Harpending 1992) and based on the complete temporal and spatial distribution of the data.

Mismatch plots from Belgium, Sweden and the whole distribution showed expansion profiles with unimodal distributions. All matched the topology shown in Fig. 3. Time since expansion was estimated at 60,000 years BP (range 710–75,000 years) with moderate and biologically sensible confidence intervals (Table 3). Similarly, female effective population sizes (*N*_*ef*_) were very large at all scales within the analysis, with levels in the west being an order of magnitude larger than the east (Table 3).

**Figure 4:**
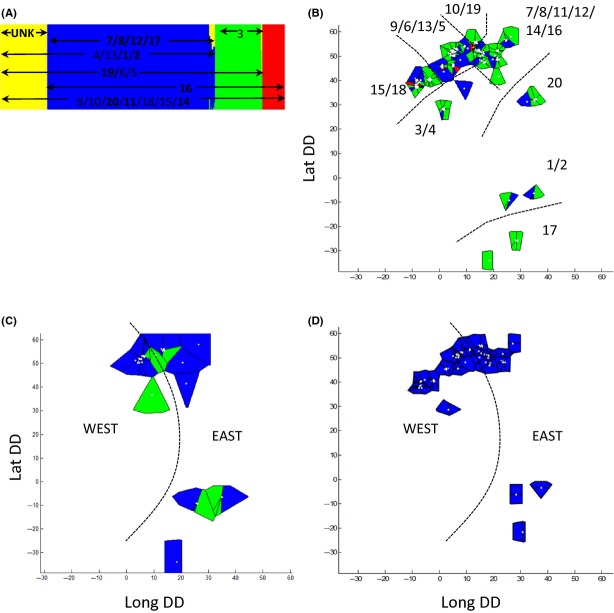
BAPS cluster analysis: (A) mtDNA clusters (*k* = 4) based on nonspatial admixture analysis. Numbers in bold represent regions within the eastern migration pool; (B) distribution of mtDNA clusters based on spatial admixture analysis (*k* = 3), including the historical and contemporary data set. The red clusters are those belonging to Network 2 in the TCS analysis (Fig. 2 – inset a). Sample locations are as follows: 1) Africa_a, 2) Africa_b, 3) Africa_c, 4) Algeria, 5) Austria, 6) Belgium, 7) Czech Republic, 8) Estonia, 9) France, 10) Germany, 11) Latvia, 12) Macedonia, 13) the Netherlands, 14) Poland, 15) Portugal, 16) Slovakia, 17) South Africa, 18) Spain, 19) Sweden, and 20) Israel.; (C) pre-reintroduction samples based on mtDNA data (*k* = 2); (D) spatial admixture analysis using microsatellite data showing *k* = 1 at the scale of the complete data set.

The BAPS admixture analysis clearly identifies *k* = 4 clusters (Fig. 4A). When including spatial information, *k* is reduced to 3, and cluster membership is distributed randomly on either side of the migratory divide (Fig. 4B). Samples belonging to the second network (Fig. 2 – inset a) belong to a single cluster present in both the east and west migration pools (Fig. 4B; red cluster). Removing these from the analysis did not change the distribution of the green and blue clusters in Fig. 4B, and analyzing the red cluster independently did not produce additional population subdivision. Analyzing the historical samples separately produces two clusters (*k* = 2; Fig. 4C) represented in both migration pools. Nevertheless, SAShA analysis indicates a very weak signature of structure (OM = 1471.7 km, expected 1690.9 km; *P* = 0.046). Jackknife analysis clearly shows that the greatest percent change in OM is due to the global distribution of haplotype CC003 (Table S4). Both CC040 and CC005 are weakly significant (Fig. S1). In each instance, these haplotypes are only found in the western regions of Belgium, France, Portugal, and Spain, were absent from the historical set, and do not form part of the second network. Rerunning the analysis with CC003 removed shows stronger evidence for overall structure (OM = 1174.8 km, expected 1729.8 km, *P* < 0.0001; Fig. S2).

### Microsatellite DNA

The amplification success across loci was on average 79, 68, and 80% for the whole, historical, and contemporary data sets, respectively. All loci were consistent with Hardy–Weinberg equilibrium expectations (*P* > 0.05) except WS*μ*03 in Algeria, France, Portugal, and Poland; WS*μ*20 in Belgium, Poland, and Spain; and WS*μ*23 in Poland and Spain. The presence of null alleles was indicated in Algeria and Latvia at WS*μ*03 (*P* < 0.05) and Portugal, Slovakia and Spain at WS*μ*20 (*P* < 0.05). Analyses were repeated with omission of one or more of these loci, without significantly altering the results. No linkage disequilibrium was detected between any pair of loci after correction for multiple testing using the Bonferroni or the less conservative B-Y FDR method.

Heterozygosity and the mean number of alleles (Table 2) showed some variation among regions and between migration pools. However, when controlling for sample size variation, there was little difference in allelic richness between migration pools in either the historical or contemporary samples (Table 2).

**Table 2:** Microsatellite diversity statistics by region, migration pool, and for the complete distribution. Belgium, the Netherlands, and Sweden contributed samples to the historical and contemporary analysis and were analyzed at each level. Regions with only one sample were excluded from “All Regions,” but included within “Migration Pool”. Data were analyzed in subsets; footnotes list those samples included in each level of the analysis. *n* = number of samples; Hexp = expected heterozygosity; Hobs = observed heterzygosity; MNA = mean number of alleles; AR = allelic richness (based on 16 samples). Values after expected and observed gene diversity are standard errors

Region	*n*	Hexp	Hobs	MNA	AR
All Regions					
East Africa (Africa_a and Africa_b)^1^	9	0.309 ± 0.347	0.316 ± 0.275	2.3	
Algeria	15	0.389 ± 0.262	0.377 ± 0.284	2.6	
Austria	5	0.372 ± 0.172	0.474 ± 0.279	2.2	
Belgium	14	0.408 ± 0.242	0.335 ± 0.232	3.2	
Belgium – historical	3	0.414 ± 0.234	0.630 ± 0.389	2.4	
Belgium – contemporary	11	0.392 ± 0.237	0.276 ± 0.234	2.8	
Czech Republic	4	0.290 ± 0.288	0.407 ± 0.426	1.9	
France	42	0.414 ± 0.207	0.410 ± 0.231	4.9	
Germany	12	0.481 ± 0.219	0.491 ± 0.241	3.4	
Latvia	8	0.505 ± 0.197	0.372 ± 0.381	3.2	
The Netherlands	27	0.429 ± 0.216	0.418 ± 0.258	3.3	
The Netherlands – historical	13	0.393 ± 0.195	0.365 ± 0.254	2.7	
The Netherlands – contemporary	14	0.438 ± 0.230	0.444 ± 0.285	2.7	
Poland	42	0.420 ± 0.204	0.387 ± 0.220	3.7	
Portugal	44	0.449 ± 0.182	0.389 ± 0.191	4.3	
Slovakia	8	0.523 ± 0.249	0.451 ± 0.313	3.1	
South Africa	3	0.354 ± 0.339	0.417 ± 0.283	2.3	
Spain	44	0.432 ± 0.210	0.373 ± 0.225	4.1	
Sweden	18	0.433 ± 0.212	0.427 ± 0.226	3.0	
Sweden – historical	7	0.427 ± 0.208	0.465 ± 0.294	2.4	
Sweden – contemporary	11	0.408 ± 0.222	0.415 ± 0.255	2.7	
Migration Pool					
Historical^2^					
East	16	0.397 ± 0.186	0.382 ± 0.199	3.1	3.1
West	16	0.416 ± 0.218	0.419 ± 0.257	2.6	2.6
Contemporary^3^					
East	76	0.457 ± 0.195	0.396 ± 0.205	5.2	3.84
West	170	0.440 ± 0.206	0.386 ± 0.207	6.0	3.78
Total sample					
East	92	0.454 ± 0.192	0.397 ± 0.204	5.1	
West	186	0.440 ± 0.207	0.389 ± 0.210	6.1	
Complete distribution	295	0.449 ± 0.201	0.398 ± 0.202	7.0	

1 This sample represents the historical population. There were no contemporary samples available for this region.

2 Regions represented are as follows: *East* – East Africa *n* = 9, Sweden *n* = 7; *West* – Belgium *n* = 3, the Netherlands *n* = 13.

3 Samples (*n* = 17) from Austria and Germany not included as they may belong to either migration pool. Regions represented are as follows: *East* – Czech Rep *n* = 4, Latvia *n* = 8, Poland *n* = 42, Slovakia *n* = 8, South Africa *n* = 3, Sweden *n* = 11; *West* – Algeria *n* = 15, Belgium *n* = 11, France *n* = 42, the Netherlands *n* = 14, Portugal *n* = 44, Spain *n* = 44.

Tests for recent expansion in the microsatellite data using BOTTLENECK (one tailed Wilcoxon test for H deficiency) and the SMM and TPM models were significant in the western (*P* = 0.00684 under both models) as well as in the eastern pool (*P* = 0.0098 and 0.0137, respectively) and for the combined data set (*P* = 0.00098 and 0.00195, respectively). No significant deviations were detected under the IAM (*P* > 0.05).

**Figure 5:**
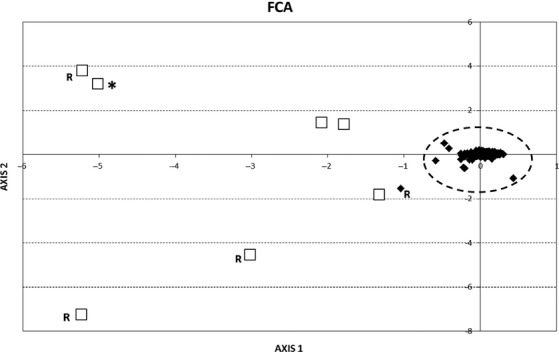
Factor correspondence analysis based on 9 microsatellite loci. The main cluster (within the dotted circle) describes over 97% of the data. Outlier individuals are from Portugal (□) and France (♦). Those with an R belong to mtDNA Network 2 (Fig. 2 – inset a). The individual with an asterisk corresponds to mtDNA haplotype CC040 and was identified in the SAShA analysis as contributing to a weak signature of structure in the western migration pool.

Both STRUCTURE and BAPS indicated the highest likelihood value for *k* = 1 (prob. = 0.99), in both the historical and contemporary data sets (Fig. 4D). This absence of structure was visually obvious in the FCA, which showed one homogeneous cluster of individuals, with the exception of 7 outliers with Portuguese and French origin (Fig. 5). This was consistent with AMOVA (F_*CT*_ 0.0009, F_*ST*_ 0.005; *P* > 0.05) and D_*est*_ (0.001; *P* > 0.05) estimates.

## Discussion

We were specifically interested in identifying two patterns in the data: geographically and genetically defined white stork populations, and genetic homogenization as a direct consequence of translocation activity. Given what is known from census information about dramatic population declines and regional extinctions (NABU 2006), we had also expected to see a strong signature of genetic bottleneck, at least among western regions.

In fact, the data showed a surprising lack of structure at any spatial or temporal scale, and diversity levels were remarkably comparable within marker sets, suggesting that even though birds were moved between migration flyways, the impact of this has been negligible, given that there is evidence of admixture prior to translocation activities. The current population of European white stork are effectively panmictic at both mitochondrial and nuclear markers based on the current analysis. Within the mtDNA data set, it is clear that the current lack of structure is driven by the blanket distribution of the most common haplotype, although notably the SaShA analysis detected some degree of structuring with this haplotype removed. Although not quantifiable, this suggests some level of permeability between flyways, which is in accordance with unpublished ring-recovery data, and what we know about occasional moderate natal dispersal distances (Chernetsov et al. 2006; Itonaga et al. 2011).

Regions showing significant mtDNA bottlenecks (Table 1) did not show expected levels of reduced diversity relative to the total sample. This suggests that population recovery may have been rapid and assisted by high levels of *N*_*e*_*,* or homogenizing gene flow between geographic populations. Storks are reproductively mature between two and seven years and potentially very long-lived (>30 years; Cramp et al. 1977; Euring 2010). It has been shown that species with long generation times, and medium to large effective population sizes that have undergone a rapid expansion (as indicated by the mismatch topology for this species; Fig. 3; Rogers and Harpending 1992), are naturally buffered against the loss of genetic diversity (Hailer et al. 2006; Lippé et al. 2006; Arenas et al. 2012).

In general, the timing of expansion signatures (average: 60,000 years) bore no relationship to modern translocation activities, or population reductions of the early 20th century (Table 3). This indicates that east and west flyways were admixed long before artificial mixing associated with reintroduction events and that detected bottlenecks in the mtDNA data are likely due to much earlier events. The existence of three spatial clusters in the BAPS mtDNA analysis shows a deep evolutionary structure. However, separation to distinct geographically defined mtDNA lineages appears impossible without the addition of much older, if not ancient DNA material. Serial expansion profiles and a progressive evolution of new haplotypes with each expansion node in the network (Fig. 2; Avise 2000) may be the result of glaciation cycles as has been suggested for similarly distributed European bird species (Hagemeijer and Blair 1997; Avise and Walker 1998), but requires further investigation. The link between the nonspatial clustering of haplotypes in BAPS (Fig. 4A) and the TCS network is shown in Fig. 2, where colored boxes, numbered one to four, show that those haplotypes belonging to a particular BAPS cluster are more closely “related” to one another in the network, than to those assigned to a different cluster. Box color matches the cluster color in Fig. 4A. These lineages are clearly distributed across the current range, but it does confirm that at some stage in the very distant past that there was sufficient population separation to produce distinct lineages.

**Table 3:** Mismatch statistics including time since expansion and female effective population size (*N*_*ef*_), where *r* = the raggedness index showing the probability that the curve is more or less ragged than expected under the sudden expansion model; tau = units of mutational time; theta 1 = a correlate of final population size (Rogers and Harpending 1992; Excoffier and Lischer 2010)

Spatial and Temporal Group	*r* (P(Sim rag ≥ Obs rag))	tau (95% C.I.)	theta 1 (95% C.I.)	Time Since Expansion (95% C.I.)	Nef (95% C.I.)
Belgium	0.027 (0.54)	4.34 (1.83–7.07)	17.697 (7.99–99,999)	58,000 (24,000–94,000)	118,612 (53,538–670M)
Belgium – historical only	0.077 (0.7)	2.781 (0.77–4.92)	99,999 (5.97–99,999)	37,000 (10,000–66,000)	670M (40,033–670M)
Sweden	0.049 (0.56)	5.666 (0.44–15.23)	5.488 (3.05–99,999)	75,000 (6000–204,000)	36,782 (20,442–670M)
East Mig Pool – historical only	0.041 (0.6)	4.008 (0.84–7.71)	6.265 (3.32–99,999)	53,000 (11,000–103,000)	41,990 (22,245–670M)
West Mig Pool – historical only	0.029 (1.0)	0.053 (0.0–0.59)	99,999 (99,869–99,999)	710 (0–8000)	670M (669M–670M)
East Mig Pool	0.023 (0.79)	4.521 (0.9–8.33)	5.552 (3.18–99,999)	60,000 (12,000–111,000)	37,211 (21,300–670M)
West Mig Pool	0.148 (0.8)	4.406 (1.52–7.61)	9.668 (5.26–99,999)	59,000 (20,000–102,000)	64,798 (35,247–670M)
Total Sample	0.014 (0.84)	4.539 (1.27–7.69)	7.859 (4.48–99,999)	60,000 (20,000–102,000)	52,674 (29,993–670M)

M, Million; Nef, effective female population size.

The separation of the Network 2 group by TCS (Fig. 2 – inset a) is difficult to explain. Haplotypes in this cluster were found in both flyways and only in the contemporary group. These individuals were sequenced with forward and reverse primers and showed an average of 84.5% concordance with the reference sequence and only 77.8% concordance with the most closely related species, *C. boyciana*. The fact that they were only in the contemporary set may simply be a sample size artifact, particularly given the position of haplotypes 34 and 63 in the main network, both of which were from the historical sample. A similar anomaly in the microsatellite data shows seven individuals sampled in Portugal, and one from France, separate from the main FCA cluster (Fig. 5). Half of these individuals display mtDNA profiles from Network 2. But again, additional work needs to be performed to explain this.

The loss of some haplotypes between the historical and contemporary data sets may reflect stochastic processes linked to recent short-term population crashes in Europe as a result of climate or anthropogenic influence (Oppenheimer 2003; Luterbacher et al. 2004). The average age of historical samples was only 100 years, so we do not think that haplotype loss is a function of miscoding lesions (*sensu* Mateiu and Rannala 2008), and common base pair changes normally associated with miscoding lesions occurred with equal or greater frequency in the contemporary sample (Table S2).

### Looking toward the future

The fact that this species appears to have suffered no ill effect following the opportunistic translocation of individuals makes it a lucky exception. However, in light of our results, the reproductive failures among the Swedish founder population remain unexplained (Olsson 2007). It may be that the poor reproduction in the reintroduced population is a result of generations of captive breeding, or failure to adapt locally, and highlights the fact that non-neutral markers may be important in determining stock suitability prior to translocation or reintroduction.

The Network 2 haplotypes and outlier alleles in the microsatellite FCA are very intriguing. An expanded sample set may provide more context to explain this, and we are currently investigating this.

This study is the first to look at the phylogeographic history of the European white stork, a species that has evolved as an important flagship to highlight environmental problems and specifically habitat alteration and human land-use impacts throughout Europe (Olsson and Rogers 2009; Kaługa et al. 2011). It is an important step in planning future conservation management of this widespread species, particularly following on from intensive reintroduction efforts and ongoing supplementary feeding associated with some programs. In addition, human intervention in the form of rubbish dumps as feeding sites (specifically in the Iberian region), the provision of artificial nest platforms to offset the loss of natural nesting areas, and the impacts of power pylon nesting (Kaługa et al. 2011) have all contributed to a positive trend of population increase throughout the species range. The number of breeding pairs is reported to have shown an 89% and 28% increase in the west and east, respectively, since the 1994 census, with the European breeding population having increased to 210,000 pairs (NABU 2006), and expected to show a further increase by the 2014 census (Thomsen, NABU; pers. comm.). It is unclear what impact the removal of external support would have on population numbers given that habitat loss and modification is likely to continue. However, overall storks have shown themselves to be a highly adaptable species. The high retention of genetic diversity, high *N*_*ef*_*,* and apparent absence of recent genetic bottleneck associated with early 20th-century declines suggest that the species is well equipped to respond to future environmental pressures and lend some weight to the argument (e.g., Schaub et al. 2004) that management should be removed under long-term monitoring conditions, with effort transferred to habitat rehabilitation instead.

## Data Accessibility

DNA sequences: Genbank Accession Nos JN410952-JN411056; JN411057, and JN411058. Microsatellite data: The following files have been submitted to the DRYAD system(http://doi.org/10.5061/dryad.jm361): 1). Stork2 for DRYAD.xls = all data (295 indiv × 9 loci), 2) Stork3 for DRYAD.xls = only data on Contemporary samples (263 × 9 loci), and 3) StorkMuseum for DRYAD.xls = only data on Historical samples (32 × 9 loci). Further details on samples: see Supporting Information.

## Supporting Information

**Data S1.** Additional analysis of interest and not included in the manuscript.

**Table S1.** Museum sample information and museum accession codes. Sequence labels and mtDNA haplotype designations refer to the current study (*n* = 54).

**Table S2.** Variable sites among unique haplotypes for Ciconia ciconia across *n* = 373 bp of control region.

**Table S3.** Count of the regional distribution of unique haplotypes and their Genbank Accession Numbers. Haplotype codes in bold were found in the Historical sample set.

**Table S4.** SAShA jackknife results for all alleles including the most common (CC03) seen here as Allele 1.

**Figure S1.** a) Observed (OM) versus expected mean (EM) distribution of alleles when considering all haplotypes. b) Haplotype-by-haplotype analysis of the complete *C. ciconia* data set. The number of occurrences of each haplotype (*n*) and significance level (*P*) are given along with the haplotype specific SAShA statistics. The expected distribution is represented with circles and the observed by triangles. Allele 8 (CC040) and Allele 9 (CC005) show significant aggregation. Geographic distance (km) and Cumulative frequency of alleles describe the x and y axis, respectively.

**Figure S2.** Observed versus expected mean distribution of alleles with the most common haplotype (CC003) removed showing that the global distribution of CC003 overshadows some degree of underlying population structure.
