# Supplementary material for: Is population structure in the European white stork determined by flyway permeability rather than translocation history?
Source: Ecol Evol. 2013 Nov 7;3(15):4881–95. doi: 10.1002/ece3.845 (PMC3892355; doi:10.1002/ece3.845)

## Supporting Information

**Shephard J, Ogden R, Tryjanowski P, Olsson O, Galbusera P** 2013 Is population structure in the European white stork determined by flyway permeability rather than translocation history?

Mitochondrial DNA Analysis

SAMOVA

To avoid artificially aggregating regions for analysis we used SAMOVA version 1.0 (Dupanloup *et al.* 2002), to define group structure. SAMOVA uses a spatial analysis of variance to maximise the variance among groups ($F_{CT}$) for a given value of $k$. Analyses were run for Historical and Contemporary samples independently and combined based on 100 initial conditions and 10000 steps for values of $k$ = 2 to $k$ = 10. Defined groups bore no resemblance to a geographic split among migration pools at any temporal scale. That is, defined groups were randomly comprised of regions from both sides of the migration divide. No other logical spatial partitioning of the data was found.

Microsatellite Analysis

2MOD

Deviation from migration/drift equilibrium was tested by comparing the relative likelihoods of 'geneflow/drift' and 'drift only' models with the program 2MOD, using a MCMC procedure with $10^5$ iterations and a burn-in of $10^4$ (Ciofi *et al.* 1999). Time intervals between population founding and population sampling were assumed sufficiently short such that effects of mutations were negligible (drift only), while mutation rates were assumed much smaller than migration rates (geneflow/drift). Bayes factors were calculated to infer decisive

power estimates of the most likely models (Goodman 1999; Jeffreys 1961). The results indicated equilibrium between gene flow and drift. Bayes factors were 1.4 (weak support) and 142 (very strong support) for Historical and Contemporary samples, respectively.

## References


Ciofi C, Beaumont M, Swingland I, Bruford M (1999) Genetic divergence and units for conservation in the Komodo Dragon *Varanus komodoensis*. *Proceedings of the Royal Society of London Series B-Biological Sciences*, **266**, 2269-2274.

Dupanloup I, Schneider S, Excoffier L (2002) A simulated annealing approach to define the genetic structure of populations. *Molecular Ecology*, **11**, 2571-2581.

Goodman SN (1999) Toward Evidence-Based Medical Statistics. 2: The Bayes Factor. *Annals of Internal Medicine*, **130**, 1005-1013.

Jeffreys H (1961) *Theory of probability third edition*, Third edn. Oxford University Press, Oxford.


## Supporting Tables

Table S1 Museum sample information and museum accession codes. Sequence labels and mtDNA haplotype designations refer to the current study (*n* = 54).

Table S2 Variable sites among unique haplotypes for Ciconia ciconia across *n* = 373bp of control region.

Table S3 Count of the regional distribution of unique haplotypes and their Genbank Accession Numbers. Haplotype codes in bold were found in the Historical sample

set. CC011 and CC016, marked with an asterix, were the only haplotypes found in both the Historical and Contemporary dataset

Table S4 SAShA jackknife results for all alleles including the most common (CC03) seen here as Allele 1. Alleles correspond to haplotypes ranked according to frequency. Each row represents an analysis with each allele removed sequentially. The test statistic OM, describes the observed mean distance between alleles. Where OM is less than the expected mean (EM), alleles are considered to be aggregated (under-distributed). Where OM is larger than EM, alleles are considered overdispersed (panmictic). A jackknifing procedure allows the identification of which alleles contribute to the distribution.

**Supporting Figures**

Fig. S1 a) Observed (OM) versus expected mean (EM) distribution of alleles when considering all haplotypes. b) Haplotype-by-haplotype analysis of the complete *C.ciconia* dataset. The number of occurances of each haplotype (*n*) and significance level (*p*) are given along with the haplotype specific SAShA statistics. The expected distribution is represented with circles and the observed by triangles. Allele 8 (CC040) and Allele 9 (CC005) show significant aggregation. Geographic distance (km) and Cumulative frequency of alleles describe the x and y axis respectively.

Fig. S2 Observed versus expected mean distribution of alleles with the most common haplotype (CC003) removed showing that the global distribution of CC003 overshadows some degree of underlying population structure.

**Table S1 Museum sample information and museum accession codes. Sequence labels and mtDNA haplotype designations refer to the current study (N=54)**

| Species | Country of Holding | Location of Holding | Source Location | Birth Country | Suggested Mig Pool | Accession No. | Accession Year | Latitude DD | Longitude DD | Sample Type | Sequence Label | mtDNA Haplotype | GenBank Accession No |
|---|---|---|---|---|---|---|---|---|---|---|---|---|---|
| Ciconia ciconia | Belgium | Brussels Natural History Museum | Africa,Belgian Congo-Nat Park Upenba | Africa | East | BNHM55752 | 1946 | -9.1999 | 25.8500 | Dried Toe Pad | AFR_O_01 | CC056 | JN411006 |
| Ciconia ciconia | Belgium | Brussels Natural History Museum | Africa,Belgian Congo-Nat Park Nawangolu Uele | Africa | East | BNHM22.577 | 1948 | -9.1999 | 25.8500 | Dried Toe Pad | AFR_O_02 | CC056 | JN411006 |
| Ciconia ciconia | Belgium | Brussels Natural History Museum | Africa, Belgian Congo-Tanganika (Musosa) | Africa | East | BNHM20578 | 1941 | -9.1999 | 25.8500 | Dried Toe Pad | AFR_O_03 | CC057 | JN411007 |
| Ciconia ciconia | Belgium | Brussels Natural History Museum | Africa, Belgian Congo-Tanganika (Musosa) | Africa | East | BNHM20577 | 1942 | -9.1999 | 25.8500 | Dried Toe Pad | AFR_O_04 | CC058 | JN411008 |
| Ciconia ciconia | Belgium | Brussels Natural History Museum | Belgium, Knokke | Belgium | West | BNHM449256 | 1931 | 51.3500 | 3.2667 | Dried Toe Pad | BE_O_223 | CC010 | JN410960 |
| Ciconia ciconia | Belgium | Brussels Natural History Museum | Belgium, Liege | Belgium | West | BNHM449257 | ? | 50.6333 | 5.5667 | Dried Toe Pad | BE_O_224 | CC011 | JN410961 |
| Ciconia ciconia | Belgium | Brussels Natural History Museum | Belgium | Belgium | West | BNHM449258 | 1932 | 50.8333 | 4.3333 | Dried Toe Pad | BE_O_225 | CC012 | JN410962 |
| Ciconia ciconia | Belgium | Brussels Natural History Museum | Belgium, Alsemberg | Belgium | West | BNHM451409 | 1935 | 50.7333 | 4.3167 | Dried Toe Pad | BE_O_226 | CC013 | JN410963 |
| Ciconia ciconia | Belgium | Brussels Natural History Museum | Belgium, Alsemberg | Belgium | West | BNHM451410 | 1935 | 50.7333 | 4.3167 | Dried Toe Pad | BE_O_227 | CC014 | JN410964 |
| Ciconia ciconia | Belgium | Brussels Natural History Museum | Belgium, Saint Gerard | Belgium | West | BNHM451456 | ? | 50.3500 | 4.7500 | Dried Toe Pad | BE_O_228 | CC015 | JN410965 |
| Ciconia ciconia | Belgium | Brussels Natural History Museum | Belgium, Brussels | Belgium | West | BNHM451792 | 1829 | 50.8333 | 4.3333 | Dried Toe Pad | BE_O_229 | CC016 | JN410966 |
| Ciconia ciconia | Belgium | Brussels Natural History Museum | France, Cerfontaine | France | West | BNHM453414 | 1946 | 50.2667 | 4.0333 | Dried Toe Pad | FR_O_056 | CC011 | JN410961 |
| Ciconia ciconia | Belgium | Brussels Natural History Museum | Lüneburg, Germany | Germany | East | Helgoland208011 | 1931 | 53.3667 | 10.4833 | Dried Toe Pad | GR_022 | CC106 | JN411056 |
| Ciconia ciconia | Belgium | Brussels Natural History Museum | Russia, Rositten | Russia | East | BNHM450894 | 1935 | 50.4000 | 20.4333 | Dried Toe Pad | POL_O_64 | CC031 | JN410981 |
| Ciconia ciconia | Germany | Museum für Naturkunde, Berlin | Tansania | Africa | East | 2000/15789 | 1895-1910 | -6.3688 | 34.8888 | Dried Toe Pad | AFR_O_05 | CC058 | JN411008 |
| Ciconia ciconia | Germany | Museum für Naturkunde, Berlin | Tansania | Africa | East | 2000/15791 | 1909 | -6.3688 | 34.8888 | Dried Toe Pad | AFR_O_06 | CC059 | JN411009 |
| Ciconia ciconia | Germany | Museum für Naturkunde, Berlin | Ufipa, Tanzania | Africa | East | 2000/15793 | 1909 | -6.3688 | 34.8888 | Dried Toe Pad | AFR_O_07 | CC060 | JN411010 |
| Ciconia ciconia | Germany | Museum für Naturkunde, Berlin | Ufipa, Tanzania | Africa | East | 2000/15794 | 1909 | -6.3688 | 34.8888 | Dried Toe Pad | AFR_O_08 | CC058 | JN411008 |
| Ciconia ciconia | Germany | Museum für Naturkunde, Berlin | Ufipa, Tanzania | Africa | East | 2000/15796 | 1909 | -6.3688 | 34.8888 | Dried Toe Pad | AFR_O_09 | CC011 | JN410961 |
| Ciconia ciconia | Germany | Museum für Naturkunde, Berlin | Tansania | Africa | East | 2000/15797 | 1903 | -6.3688 | 34.8888 | Dried Toe Pad | AFR_O_10 | CC061 | JN411011 |
| Ciconia ciconia | Germany | Museum für Naturkunde, Berlin | Tansania | Africa | East | 2000/15798 | 1903 | -6.3688 | 34.8888 | Dried Toe Pad | AFR_O_11 | CC062 | JN411012 |
| Ciconia ciconia | Germany | Museum für Naturkunde, Berlin | Hellenurme | Estonia | East | 2000/15790 | 1895 | 58.1361 | 26.3899994 | Dried Toe Pad | EST_O_1 | CC031 | JN410981 |
| Ciconia ciconia | Germany | Museum für Naturkunde, Berlin | Malchow | Germany | West/East | 1990/9 | Unk | 53.4667 | 12.4167 | Dried Toe Pad | GR_017 | CC068 | JN411018 |
| Ciconia ciconia | Germany | Museum für Naturkunde, Berlin | Miletkova | Macedonia | East | 2000/15795 | 1917 | 41.6074 | 21.7449 | Dried Toe Pad | MAC_O_001 | CC011 | JN410961 |
| Ciconia ciconia | Netherlands | Naturalis - Natural History Museum, Leiden | Beja, Tunisia | Africa | West | NNHM19574 | 1951 | 36.7333 | 9.1833 | Dried Toe Pad | AFR_O_12 | CC063 | JN411013 |
| Ciconia ciconia | Netherlands | Naturalis - Natural History Museum, Leiden | s Hertongenbosch | Netherlands | West | NNHM545/CAT13 | 1906 | 51.7000 | 5.3167 | Dried Toe Pad | NL_O_05 | CC011 | JN410961 |
| Ciconia ciconia | Netherlands | Naturalis - Natural History Museum, Leiden | s Hertongenbosch | Netherlands | West | NNHM545/CAT14 | 1906 | 51.7000 | 5.3167 | Dried Toe Pad | NL_O_06 | CC011 | JN410961 |
| Ciconia ciconia | Netherlands | Naturalis - Natural History Museum, Leiden | Slikkerveer | Netherlands | West | NNHM4875 | ? | 51.8833 | 4.6167 | Dried Toe Pad | NL_O_07 | CC011 | JN410961 |
| Ciconia ciconia | Netherlands | Naturalis - Natural History Museum, Leiden | Anholt | Netherlands | West | NNHM7103 | 1910 | 52.7833 | 6.4167 | Dried Toe Pad | NL_O_08 | CC011 | JN410961 |
| Ciconia ciconia | Netherlands | Naturalis - Natural History Museum, Leiden | Assen | Netherlands | West | NNHM9141 | 1939 | 53.0000 | 6.5500 | Dried Toe Pad | NL_O_09 | CC017 | JN410967 |
| Ciconia ciconia | Netherlands | Naturalis - Natural History Museum, Leiden | Huizum | Netherlands | West | NNHM13340 | 1947 | 53.2000 | 5.8000 | Dried Toe Pad | NL_O_10 | CC012 | JN410962 |
| Ciconia ciconia | Netherlands | Naturalis - Natural History Museum, Leiden | Veenwouden | Netherlands | West | NNHM15356 | 1950 | 53.2333 | 6.0000 | Dried Toe Pad | NL_O_11 | CC011 | JN410961 |
| Ciconia ciconia | Netherlands | Naturalis - Natural History Museum, Leiden | Driebergen | Netherlands | West | NNHMCAT9 | 1865 | 52.0500 | 5.2833 | Dried Toe Pad | NL_O_12 | CC011 | JN410961 |
| Ciconia ciconia | Netherlands | Naturalis - Natural History Museum, Leiden | Ausgustinusga | Netherlands | West | NNHM24306 | 1956 | 53.2167 | 6.1667 | Dried Toe Pad | NL_O_13 | CC011 | JN410961 |
| Ciconia ciconia | Netherlands | Naturalis - Natural History Museum, Leiden | Wesermundung | Netherlands | West | NNHM24703 | 1891 | 53.2833 | 5.5667 | Dried Toe Pad | NL_O_14 | CC011 | JN410961 |
| Ciconia ciconia | Netherlands | Naturalis - Natural History Museum, Leiden | Drachten | Netherlands | West | NNHM15344 | 1950 | 53.1000 | 6.1000 | Dried Toe Pad | NL_O_15 | CC011 | JN410961 |
| Ciconia ciconia | Netherlands | Naturalis - Natural History Museum, Leiden | Schoonhoven | Netherlands | West | NNHM25017 | 1957 | 51.9500 | 4.8500 | Dried Toe Pad | NL_O_16 | CC011 | JN410961 |
| Ciconia ciconia | Netherlands | Naturalis - Natural History Museum, Leiden | Papendrecht | Netherlands | West | NNHM43102 | 1959 | 51.8333 | 4.6833 | Dried Toe Pad | NL_O_17 | CC011 | JN410961 |
| Ciconia ciconia | Netherlands | Naturalis - Natural History Museum, Leiden | Oegstgeest | Netherlands | West | NNHMCAT8 | 1865 | 52.1833 | 4.4667 | Dried Toe Pad | NL_O_18 | CC012 | JN410962 |
| Ciconia ciconia | Netherlands | Naturalis - Natural History Museum, Leiden | Sassenheim | Netherlands | West | NNHMCAT3 | 1859 | 52.2167 | 4.5333 | Dried Toe Pad | NL_O_19 | CC011 | JN410961 |
| Ciconia ciconia | Netherlands | Naturalis - Natural History Museum, Leiden | South Holland | Netherlands | West | NNHMCAT209-1 | 1851 | 52.1500 | 4.5000 | Dried Toe Pad | NL_O_20 | CC011 | JN410961 |
| Ciconia ciconia | Netherlands | Naturalis - Natural History Museum, Leiden | Leiden | Netherlands | West | NNHMCAT209-2 | 1866 | 52.1500 | 4.5000 | Dried Toe Pad | NL_O_21 | CC018 | JN410968 |
| Ciconia ciconia | Netherlands | Naturalis - Natural History Museum, Leiden | Latakoe | South Africa | East | NNHMCAT16 | 1834 | -33.9167 | 18.4167 | Dried Toe Pad | SAF_O_01 | CC011 | JN410961 |
| Ciconia ciconia | Sweden | Museum Zoology, Lund University | Råby, Lund | Sweden | East | L846/30313 | 1846 | 55.6854 | 13.2084 | Dried Toe Pad | SW_SW_O_14 | CC011 | JN410961 |
| Ciconia ciconia | Sweden | Museum Zoology, Lund University | Unknown | Sweden | East | L848/6133 | 1848 | ? | ? | Dried Toe Pad | SW_SW_O_15 | CC011 | JN410961 |
| Ciconia ciconia | Sweden | Museum Zoology, Lund University | Trollenäs | Sweden | East | L871/3009 | 1871 | 55.8659 | 13.2444 | Dried Toe Pad | SW_SW_O_16 | CC016 | JN410966 |
| Ciconia ciconia | Sweden | Museum Zoology, Lund University | Fjelie | Sweden | East | L872/3011 | 1872 | 55.7273 | 13.1047 | Dried Toe Pad | SW_SW_O_17 | CC034 | JN410984 |
| Ciconia ciconia | Sweden | Museum Zoology, Lund University | Bergsjöholm | Sweden | East | L876/3038 | 1876 | 55.4513 | 13.7773 | Dried Toe Pad | SW_SW_O_18 | CC017 | JN410967 |
| Ciconia ciconia | Sweden | Museum Zoology, Lund University | Stehag | Sweden | East | L877/3051 | 1877 | 55.9015 | 13.4004 | Dried Toe Pad | SW_SW_O_19 | CC035 | JN410985 |
| Ciconia ciconia | Sweden | Museum Zoology, Lund University | Skabersjö | Sweden | East | L879/3018 | 1879 | 55.5401 | 13.1496 | Dried Toe Pad | SW_SW_O_20 | CC011 | JN410961 |
| Ciconia ciconia | Sweden | Museum Zoology, Lund University | Farhult | Sweden | East | L909/3500 | 1909 | 56.2141 | 12.7124 | Dried Toe Pad | SW_SW_O_21 | CC011 | JN410961 |
| Ciconia ciconia | Sweden | Museum Zoology, Lund University | Skårby | Sweden | East | L925/3032 | 1925 | 55.4866 | 13.7132 | Dried Toe Pad | SW_SW_O_22 | CC011 | JN410961 |
| Ciconia ciconia | Sweden | Museum Zoology, Lund University | Silvåkra | Sweden | East | L948/3072 | 1948 | 55.6844 | 13.4944 | Dried Toe Pad | SW_SW_O_23 | CC036 | JN410986 |
| Ciconia ciconia | Sweden | Museum Zoology, Lund University | Hörja | Sweden | East | L954/3013 | 1954 | 56.2056 | 13.5898 | Dried Toe Pad | SW_SW_O_24 | CC011 | JN410961 |

NB. Sample BNHM450894 was sampled in Russia, but the Lat/Long places it in Poland. This may be due to geographic boundary changes associated with the World war. It was included in the Polish sample for analysis purposed. Irrespective of this it is considered to belong to the eastern migration pool so allocation to sample group is irrelevant at this scale

Table S2 – Variable sites among unique Haplotypes for *Ciconia ciconia* across *n* = 373bp of control region.

| | | | | | | | | | |
|---|---|---|---|---|---|---|---|---|---|
| | | | 11111111 | 1111111111 | 1111111111 | 1111111111 | 2222222222 | 2222233333 | 33333 |
| | 3345555566 | 6777778889 | 9900011111 | 1122333333 | 4444455555 | 6677789999 | 0111112244 | 5666600011 | 12333 |
| | 5813678925 | 7013572570 | 7903801236 | 7903023468 | 0268914589 | 1915642569 | 7015781356 | 6015812545 | 82034 |
| Concensus | CGTTTACTTC | -ACTATCAAA | GGCAAGCGTA | TAAT-TTTGC | GAAGTGTTGC | TGGCTTAGGC | GGGAAATCAA | GGGTGGATAA | GAGAC |
| CC001 | .......... | -........G | ..T....... | ...C-..... | .......... | .......... | ........G. | .......C.. | ..... |
| CC002 | .......... | -........G | .......... | ....A..... | .....A.... | .......... | .......... | .......C.. | ..... |
| CC003 | .......... | -......... | .......... | ....-..... | .........T | .......... | ...G...... | .......... | ..... |
| CC004 | .......... | -........G | .......... | .G..-..... | .......... | .......... | .......... | .......... | ..... |
| CC005 | .......... | -........G | .......... | ....-..... | .....A.... | .......... | .......... | .......C.. | ..... |
| CC006 | .......... | -........G | ..T....... | ....-..... | .......... | .......... | ........G. | .......... | ..... |
| CC007 | .......... | -........G | .......... | ....-..... | .......... | .......A.. | .......... | .......... | ..... |
| CC008 | .......... | -......... | .......... | ....-..... | .........T | .......... | ...G...... | .......C.. | ..... |
| CC009 | .......... | -........G | .......... | ....-..... | .......... | .......... | ........G. | .......... | ..... |
| CC010 | .......... | -G........ | ..T....... | ....-..... | .........T | .......... | .......... | .......... | ..... |
| CC011 | .......... | -......... | ..T....... | ....-..... | .........T | .......... | ...G...... | .......... | ..... |
| CC012 | .......... | -......... | ..T....... | ....-....T | .........T | .......... | ...G...... | .......... | ..... |
| CC013 | .......... | -......... | ..T....... | ....-..... | .........T | ....C..... | ...G...... | .....A.... | ..... |
| CC014 | .......... | -......... | ..T....... | ....-..... | .........T | ....C..... | ...G...... | .......... | ..... |
| CC015 | .......... | -......... | ..T....... | ....-..... | ...A.....T | .......... | ...G...... | .AA....... | ..... |
| CC016 | .......... | -......... | ..T....... | ....-..... | .........T | .......... | .......... | .......... | ..... |
| CC017 | .......... | -........G | ..T....... | ....-..... | .......... | .......A.. | .......... | .......... | ..... |
| CC018 | .......... | -......... | ..T....... | ....-..... | .........T | .......... | ...G...... | .......... | A.... |
| CC019 | .......... | -........G | .......... | ....-..... | .......... | .......... | .......... | .......... | ..... |
| CC020 | .......... | -......... | .......... | ....-..... | .........T | .......... | .......... | .......... | ..... |
| CC021 | .AC.AGTGGT | A.TATCACT. | TATTCTTAC. | C.GCG.ACAG | C.T.A-GA.. | GT-A.AG... | .......... | ........G. | ....T |
| CC022 | .......... | -........G | .......... | ....-..... | .......... | .......... | .......... | .......C.. | ..... |
| CC023 | .......... | -......... | .......... | ....-..... | .......... | .......... | ...G...... | .......... | ..... |
| CC024 | .......... | -........G | .......... | .G..-..... | .......... | .......... | .......... | ...C...... | ..... |
| CC025 | .......... | -......... | .......... | ....-..... | .........T | .......... | .......... | .......C.. | ..... |
| CC026 | .......... | -......... | .........G | ....-..... | .........T | .......... | ...G...... | .......... | ..... |
| CC027 | .......... | -........G | .......... | ....-..... | .........T | .......... | ...G...... | .......... | ..... |
| CC028 | .AC.AGTGGT | A.TATCACT. | TATTCTTAC. | C.GCG.ACAG | C.T.A-GA.. | GT-A.AG... | .........G | .......C.. | ..... |
| CC029 | .......... | -......... | .......... | ....-C.... | .........T | .......... | ...G...... | .......... | ..... |
| CC030 | .......... | -......... | .......... | ....-..... | .........T | ......G... | ...G...... | .......... | ..... |

| | | | | | | | | | |
|---|---|---|---|---|---|---|---|---|---|
| CC031 | .......... | -......... | ..T....... | ....-..... | .........T | .......... | ...G...... | .....A.... | ..... |
| CC032 | .......... | -........G | .......... | ...C-..... | .......... | .......... | .......... | .........G | ..... |
| CC033 | .......... | -......... | .......... | ....-..... | .........T | .A........ | ...G...... | .......... | ..... |
| CC034 | .......... | -........G | ..T....... | .G..-..... | .......... | .......... | .......... | ...C...... | ..... |
| CC035 | .......... | -......... | ..T....... | ....-..... | .........T | .......... | ...G...... | .......C.. | ..... |
| CC036 | .......... | -......... | ..T....... | ....-..... | .........T | .........T | ...G...... | .......... | ..... |
| CC037 | .......... | -......... | .......... | ....A..... | .........T | .......... | ...G...... | .......... | ..... |
| CC038 | .......... | -......... | .......... | ....-..... | .........T | ......G... | A..G...... | .......... | ..... |
| CC039 | .......... | -......... | .......... | ....-..... | .G.......T | .......... | ..AG..C... | .......... | ..... |
| CC040 | .......... | -......... | .......... | ...C-..... | .........T | .......... | .......... | .......... | ..... |
| CC041 | .......... | -......... | ..T....... | ...C-..... | .........T | .......... | .......... | .......... | ..... |
| CC042 | .AC.AGTGGT | A.TATCACT. | TATTCTTAC. | C.GCG.ACAG | C.T.A-GA.. | GT-A.AG... | .......... | .......... | ..... |
| CC043 | .......... | -........G | ..T....... | .G..-..... | .......... | ......G... | .......... | .......... | ..... |
| CC044 | .......... | -........G | .......... | .G..-..... | .......... | ......G... | .......... | .......... | ..... |
| CC045 | .AC.AGTGGT | A.TATCACT. | TATTCTTAC. | C.GCG.ACAG | C.T.A-GA.. | GT-A.A.... | .......... | .......... | ..... |
| CC046 | .......... | -......... | .......... | ....-..... | .........T | .....C.... | ...G...... | .......... | ....T |
| CC047 | .......... | -........G | .......... | .G..-..... | .......... | .......... | .......... | .......... | ....T |
| CC048 | .......... | -........G | ..T....... | ....-..... | .......... | .......... | .....G.T.. | .......... | ....T |
| CC049 | .......... | -........G | .......... | .G..-..... | .......... | .A........ | ...G...... | .......... | ..... |
| CC050 | .AC.AGTGGT | A.TATCACT. | TATTCTTAC. | C.GCG.ACAG | C.T.A-GA.. | GT-A.AG... | ...G...... | .......... | ....T |
| CC051 | .......... | -......... | .......... | ....-..... | .........T | .....C.... | ...G...... | .......... | ..... |
| CC052 | .AC.AGTGGT | A.TATCACT. | TATTCTTAC. | C.GCG.ACAG | C.T.A-GA.. | GT-A.A.... | .......... | .......... | .G..T |
| CC053 | .AC.AGTGGT | A.TATCACT. | TATTCTTAC. | C.GCG.ACAG | C.T.A-GA.. | GT-A.AG... | .......... | .......... | ...G. |
| CC054 | .......... | -........G | .......... | .G..-..... | .......... | .......... | .......... | .......... | .G..T |
| CC055 | .......... | -......... | ..T....... | ....-..... | .........T | .A........ | ...G...... | .......... | ..... |
| CC056 | .......... | -......... | ..T....... | ....-..... | .........T | .......... | ...G...... | .........G | ..... |
| CC057 | .......... | -........G | ..T....... | ...C-..... | .......... | .......... | .......... | ........G. | ..... |
| CC058 | .......... | -........G | ..T....... | ....-..... | .......... | .......... | .......... | .......... | ..... |
| CC059 | .......... | -........G | ..T....... | ....-..... | .......... | .......... | .......... | .......C.. | ..... |
| CC060 | .......... | -......... | ..T....... | ...C-C.... | .........T | .......... | ...G...... | .......... | A.... |
| CC061 | .......... | -........G | ..T....... | ....-..... | .......... | ......GA.. | .......... | .......... | ..... |
| CC062 | .......... | -......... | ..T....... | ....-..... | .........T | .......... | ...G...... | .......... | ....T |
| CC063 | .......... | -........G | ..T....... | .G..-..... | .......... | .......... | ........G. | ...C...... | ..... |
| CC064 | .......... | -......... | .......... | ....-..... | .........T | ..A....... | ...G...... | .......... | ..... |
| CC065 | .......... | -......... | .......... | ....-...A. | .........T | .......... | ...G...... | .......... | ..... |
| CC066 | .......... | -........G | .......... | ....-..... | .......... | ......GA.. | .......... | .......... | ..... |
| CC067 | .......... | -........G | .......T.. | .G..-..... | .....T.... | .......... | .A........ | ...C...... | ..... |
| CC068 | .AC.AGTGGT | A.TATCACT. | TATTCTTAC. | C.GCG.ACAG | C.T.A-GA.. | GT-A.AG... | ...G...... | .......... | ..... |

CC069 .......... -......... .......... ....-..... .........T ........A. ...G...... .......... .....
CC070 .AC.AGTGGT A.TATCACT. TATTCTTAC. C.GCG.ACAG C.T.A-GA.. GT-A.A.... .......... .......... ....T
CC071 .......... -......... .......... ....-..... .........T .......... .......T.. .......... .....
CC072 .......... -........G .......... ....-..... .......... .A........ .........G .......... .....
CC073 .AC.AGTGGT A.TATCACT. TATTCTTAC. C.GCG.ACAG C.T.A-GA.. GT-A.AG... .......... .......C.. .....
CC074 .......... -........G .......... .G..-..... .......... .......... .......... A......... .....
CC075 .......... -........G .......... .G..-..... .......... .......... .......... ........G. .....
CC076 .......... -......... .......... ....-..... .G.......T .......... ...G..C... .......... .....
CC077 .......... -........G .......... ....-..... .......... .......... .........G .......... .....
CC078 .......... -........G .......... .G..-..... .......... ....C..... .......... .......... .....
CC079 .AC.AGTGGT A.TATCACT. TATTCTTAC. C.GCG.ACAG C.T.A-GA.. GT-A.AG... .......... .......C.. ..A..
CC080 .......... -........G .......... ....-..... .......... .......... ......C... .......C.. .....
CC081 .ACCAGTGGT A.TATCACT. TATTCTTAC. C.GCG.ACAG C.T.A-GA.. GT-A.AG... .......T.. .......... .....
CC082 TAC.AGTGGT A.TATCACT. TATTCTTAC. C.GCG.ACAG C.T.A-GAA. GT-A.AG... .......... .......... .....
CC083 .......... -......... .......... ...C-..... .........T .A........ .......... .......... .....
CC084 .......... -........G .......... .G..-..... .......... .......... .......T.. .......... .....
CC085 .AC.AGTGGT A.TATCACT. TATTCTTAC. C.GCG.ACAG C.T.A-GA.. GT-A.AG... .......... .........G .....
CC086 .......... -......... .......... ....-..... .........T .......... ...G...... ....A..... .....
CC087 .......... -........G .......... ....-..... .....T.... .......... ......C... .......C.. .....
CC088 .......... -........G .......... .G..-..... .......... .......... ....G..... .......... ....T
CC089 .......... -........G .......... .G..-..... .......... .A........ .......... .......... .....
CC090 .......... -........G .......... ....-..... .......... .A........ .......... .......... .....
CC091 .......... -........G ..T....... ....-..... .....T.... .......... ........G. .......... .....
CC092 .AC.AGTGGT A.TATCACT. TATTCTTAC. C.GCG.ACAG C.T.A-GA.. GT-A.AG... .......... ...C...... .....
CC093 .......... -......... .......... ....-..... .....T...T .......... .......... .......... .....
CC094 .......... G-........ .......... ....-..... .........T .......... .......... .......... A....
CC095 .AC.AGTGGT A.TATCACT. TATTCTTAC. C.GCG.ACAG C.T.A-GA.. GT-A.AG... .........G .......... .....
CC096 .......... -......... .......... ...C-..... .........T ......G... A..G...... .......... .....
CC097 .......... -......... .......... ....-....T .........T .......... ...G...... .......... .....
CC098 .......... -........G .......... .G..-..... .......... .......... .......... ......G... .....
CC099 .........T -........G .......... .G..-..... .......... .......... .......... .......... .G..T
CC100 .......... -......G.. .......T.. ....-..... .....T...T .......... ...G...... .......... .....
CC101 .......... -......... .......... ....-C.... .........T .......... .......... .......... ....T
CC102 .......... -........G .......... .G..-..... .......... ......G... .......... ...C...... .....
CC103 .AC.AGTGGT A.TATCACT. TATTCTTAC. C.GCG.ACAG C.T.A-GA.. GT-A.AG... ...G...... .......... A....
CC104 .......... -........G .......... .G..-..... .......... .......... .......... ......G... A....
CC105 .......... -......... .......... ....-..... .........T .........T ...G...... .......... .....
CC106 .......... -........G ..T....... ...C-..... .......... .......... .......... .......... .....

**Table S3 Regional distribution of haplotypes. Haplotype codes in bold were found in the Historical sample set. CC011 and CC016 marked with an asterix, were the only haplotypes found in both the Historical and Contemporary dataset.**

| MtDNA Haplotype | Genbank Accession No. | Africa_a | Africa_b | Africa_c | Algeria | Austria | Belgium | Czech Republic | Estonia | France | Germany | Latvia | Macedonia | Netherlands | Poland | Portugal | Slovakia | South Africa | Spain | Sweden | Israel | Unknown | Total |
|---|---|---|---|---|---|---|---|---|---|---|---|---|---|---|---|---|---|---|---|---|---|---|---|
| CC001 | NC_002197 | | | | | | | | | | | | | | | | | | | | | 1 | 1 |
| CC002 | JN410952 | | | | | | 1 | | | | | | | | | | | | | | | | 1 |
| CC003 | JN410953 | | | | 6 | 3 | 9 | 1 | | 12 | 8 | 3 | | 7 | 25 | 7 | 3 | 2 | 28 | 4 | 4 | | 122 |
| CC004 | JN410954 | | | | | | 7 | | | 1 | | 1 | | | 4 | 11 | 1 | | 10 | | | | 35 |
| CC005 | JN410955 | | | | | | 6 | | | 3 | | | | | | | | | | | | | 9 |
| CC006 | JN410956 | | | | | | 1 | | | | | | | | | | | | | | 1 | | 2 |
| CC007 | JN410957 | | | | 1 | | 2 | | | 1 | | | | 2 | 2 | 5 | | | | | | | 13 |
| CC008 | JN410958 | | | | | | 2 | | | 4 | | | | | 3 | | 1 | | 1 | | | | 11 |
| CC009 | JN410959 | | | | | | 2 | | | | | | | | | | | | | | | | 2 |
| **CC010** | JN410960 | | | | | | 1 | | | | | | | | | | | | | | | | 1 |
| **CC011*** | JN410961 | | 1 | | | | 1 | | | 2 | 1 | | 1 | 20 | 6 | | 1 | 1 | 1 | 6 | | | 41 |
| **CC012** | JN410962 | | | | | | 1 | | | | | | | 2 | | | | | | | | | 3 |
| **CC013** | JN410963 | | | | | | 1 | | | | | | | | | | | | | | | | 1 |
| **CC014** | JN410964 | | | | | | 1 | | | | | | | | | | | | | | | | 1 |
| **CC015** | JN410965 | | | | | | 1 | | | | | | | | | | | | | | | | 1 |
| **CC016*** | JN410966 | | | | | 1 | 1 | | | | | | | | | | | | | 1 | | | 3 |
| **CC017** | JN410967 | | | | | | | | | | | | | 1 | | | | | | 1 | | | 2 |
| **CC018** | JN410968 | | | | | | | | | | | | | 1 | | | | | | | | | 1 |
| CC019 | JN410969 | | | | | 1 | 1 | | | 4 | | 1 | | 1 | 3 | 1 | | | | | | | 12 |
| CC020 | JN410970 | | | | 8 | | | | | 2 | 3 | 1 | | | 2 | 3 | | | 3 | 1 | | | 23 |
| CC021 | JN410971 | | | | | | | | | | | | | | 1 | | 1 | | | | | | 2 |
| CC022 | JN410972 | | | | | | | | | 1 | 1 | | | | 3 | | | | | | | | 5 |
| CC023 | JN410973 | | | | | | | | | | | | | | 1 | | | | | | | | 1 |
| CC024 | JN410974 | | | | | | | | | | | | | | 3 | 2 | 2 | | | 1 | | | 8 |
| CC025 | JN410975 | | | | | | | | | | | | | | 2 | | | | | | | | 2 |
| CC026 | JN410976 | | | | | | | | | | | | | | 1 | | | | | | | | 1 |
| CC027 | JN410977 | | | | | | | | | | | | | | 1 | | | | | | | | 1 |
| CC028 | JN410978 | | | | | | | | | | | | | | 1 | | | | | | | | 1 |
| CC029 | JN410979 | | | | | | | | | | | | | | 1 | 3 | | | 1 | | | | 5 |
| CC030 | JN410980 | | | | | | | | | | | | | | 1 | | | | | | | | 1 |
| **CC031** | JN410981 | | | | | | | | | | | | | | 1 | | | | | | | | 1 |
| CC032 | JN410982 | | | | | | | | | 1 | | | | | | | | | | 3 | 1 | | 5 |
| CC033 | JN410983 | | | | | | | | | | | | | | | | | | | 1 | | | 1 |
| **CC034** | JN410984 | | | | | | | | | | | | | | | | | | | 1 | | | 1 |
| **CC035** | JN410985 | | | | | | | | 1 | | | | | | | | | | | 1 | | | 2 |
| **CC036** | JN410986 | | | | | | | | | | | | | | | | | | | 1 | | | 1 |
| CC037 | JN410987 | | | | | | | | | | | | | | | | | | | 1 | | | 1 |
| CC038 | JN410988 | | | | | | | | | | | | | | | | | | | 1 | | | 1 |
| CC039 | JN410989 | | | | | | | | | | | | | | | | | | 1 | | | | 1 |
| CC040 | JN410990 | | | | | | | | | 2 | | | | | | 3 | | | 5 | | | | 10 |
| CC041 | JN410991 | | | | | | | | | 1 | | | | | | | | | 1 | | | | 2 |
| CC042 | JN410992 | | | | | | | | | 1 | | | | | | 2 | | | 1 | | | | 4 |
| CC043 | JN410993 | | | | | | | | | | | | | | | | | | 1 | | | | 1 |
| CC044 | JN410994 | | | | | | | | | 1 | | | | | | 3 | | | 4 | | | | 8 |
| CC045 | JN410995 | | | | | | | | | | | | | | | | | | 1 | | | | 1 |
| CC046 | JN410996 | | | | | | | | | | | | | | | 1 | | | 3 | | | | 4 |
| CC047 | JN410997 | | | | | | | | | | | | | | | | | | 1 | | | | 1 |
| CC048 | JN410998 | | | | | | | | | | | | | | | 2 | | | 2 | | | | 4 |
| CC049 | JN410999 | | | | | | | | | | | | | | | 5 | | | 2 | | | | 7 |
| CC050 | JN411000 | | | | | | | | | 3 | | | | | | 4 | | | 1 | | | | 8 |
| CC051 | JN411001 | | | | | | | | | | | | | | | | | | 1 | | | | 1 |
| CC052 | JN411002 | | | | | | | | | | | | | | | | | | 2 | | | | 2 |
| CC053 | JN411003 | | | | | | | | | 1 | | 1 | | | | | | | | | | | 2 |
| CC054 | JN411004 | | | | | 1 | | | | | | | | | | | | | | | | | 1 |
| CC055 | JN411005 | | | | | 1 | | | | | | | | | | | | | | | | | 1 |
| **CC056** | JN411006 | 2 | | | | | | | | | | | | | | | | | | | | | 2 |
| **CC057** | JN411007 | 1 | | | | | | | | | | | | | | | | | | | | | 1 |
| **CC058** | JN411008 | 1 | 2 | | | | | | | | | | | | | | | | | | | | 3 |
| **CC059** | JN411009 | | 1 | | | | | | | | | | | | | | | | | | | | 1 |
| **CC060** | JN411010 | | 1 | | | | | | | | | | | | | | | | | | | | 1 |
| **CC061** | JN411011 | | 1 | | | | | | | | | | | | | | | | | | | | 1 |
| **CC062** | JN411012 | | 1 | | | | | | | | | | | | | | | | | | | | 1 |
| **CC063** | JN411013 | | | 1 | | | | | | | | | | | | | | | | | | | 1 |
| CC064 | JN411014 | | | | | | | | | | | | | | | | | 1 | | | | | 1 |
| CC065 | JN411015 | | | | | | | 3 | | | | | | | | | | | | | | | 3 |
| CC066 | JN411016 | | | | | | | | | | 3 | | | | | 1 | | | | | | | 4 |
| CC067 | JN411017 | | | | | | | | | | 1 | | | | | | | | | | | | 1 |
| CC068 | JN411018 | | | | | | | | | | 1 | | | | | | | | | | | | 1 |
| CC069 | JN411019 | | | | | | | | | | 1 | | | | | | | | | | | | 1 |
| CC070 | JN411020 | | | | | | | | | | | | | | | 1 | | | | | | | 1 |
| CC071 | JN411021 | | | | | | | | | | | | | | | 2 | | | | | | | 2 |
| CC072 | JN411022 | | | | | | | | | | | | | | | 2 | | | | | | | 2 |
| CC073 | JN411023 | | | | | | | | | | | | | | | 2 | | | | | | | 2 |
| CC074 | JN411024 | | | | | | | | | | | | | | | 1 | | | | | | | 1 |
| CC075 | JN411025 | | | | | | | | | | | | | | | 1 | | | | | | | 1 |
| CC076 | JN411026 | | | | | | | | | | | | | | | 4 | | | | | | | 4 |
| CC077 | JN411027 | | | | | | | | | 1 | | | | | | 1 | | | | | | | 2 |
| CC078 | JN411028 | | | | | | | | | 1 | | | | | | 1 | | | | | | | 2 |
| CC079 | JN411029 | | | | | | | | | | | | | | | 1 | | | | | | | 1 |
| CC080 | JN411030 | | | | | | | | | | | | | | | 3 | | | | | | | 3 |
| CC081 | JN411031 | | | | | | | | | | | | | | | 1 | | | | | 2 | | 3 |
| CC082 | JN411032 | | | | | | | | | | | | | | | 2 | | | | | | | 2 |
| CC083 | JN411033 | | | | | | | | | | | | | | | 1 | | | | | | | 1 |
| CC084 | JN411034 | | | | | | | | | | | | | | | 1 | | | | | | | 1 |
| CC085 | JN411035 | | | | | | | | | 1 | | | | | | 1 | | | | | | | 2 |
| CC086 | JN411036 | | | | | | | | | 1 | | | | | | 1 | | | | | | | 2 |
| CC087 | JN411037 | | | | | | | | | 1 | | | | | | | | | | | | | 1 |
| CC088 | JN411038 | | | | | | | | | 1 | | | | | | | | | | | | | 1 |
| CC089 | JN411039 | | | | | | | | | 1 | | | | | | | | | | | | | 1 |
| CC090 | JN411040 | | | | | | | | | 1 | | | | | | | | | | | | | 1 |
| CC091 | JN411041 | | | | | | | | | 1 | | | | | | | | | | | | | 1 |
| CC092 | JN411042 | | | | | | | | | 1 | | | | | | | | | | | | | 1 |
| CC093 | JN411043 | | | | | | | | | 1 | | | | | | | | | | | | | 1 |
| CC094 | JN411044 | | | | | | | | | 2 | | | | | | | | | | | | | 2 |
| CC095 | JN411045 | | | | | | | | | 2 | | | | | | | | | | | | | 2 |
| CC096 | JN411046 | | | | | | | | | 1 | | | | | | | | | | | | | 1 |
| CC097 | JN411047 | | | | | | | | | 1 | | | | | | | | | | | | | 1 |
| CC098 | JN411048 | | | | | | | | | | | | | | | | 1 | | | | | | 1 |
| CC099 | JN411049 | | | | | | | | | | | | | | | | 1 | | | | | | 1 |
| CC100 | JN411050 | | | | | | | | | | | | | | | | 1 | | | | | | 1 |
| CC101 | JN411051 | | | | | | | | | | | | | 1 | | | | | | | | | 1 |
| CC102 | JN411052 | | | | | | 1 | | | | | | | | | | | | | | | | 1 |
| CC103 | JN411053 | | | | | | | | | | | | | | | | | | | | 1 | | 1 |
| CC104 | JN411054 | | | | | | | | | | | | | | | | | | | | 1 | | 1 |
| CC105 | JN411055 | | | | | | | | | | | | | | | | | | | | 1 | | 1 |
| **CC106** | JN411056 | | | | | | | | | | 1 | | | | | | | | | | | | 1 |
| | | 4 | 7 | 1 | 15 | 7 | 39 | 4 | 1 | 57 | 20 | 7 | 1 | 35 | 61 | 79 | 12 | 4 | 70 | 23 | 11 | | 459 |

Table S4  SAShA jackknife results for all alleles including the most common (CC03) seen here as Allele 1.

| Allele jacknifed | % of Data | OM | EM | *P* | % Change in OM |
|---|---|---|---|---|---|
| 0 | - | 1471.7 | 1690.9 | 0.046 | 0.000 |
| **1** | **26.64** | **1174.8** | **1690.9** | **0** | **-20.172** |
| 2 | 8.95 | 1491 | 1690.1 | 0.11 | 1.314 |
| 3 | 7.64 | 1495 | 1690.9 | 0.13 | 1.583 |
| 4 | 5.02 | 1466.8 | 1690.9 | 0.055 | -0.337 |
| 5 | 2.84 | 1472.8 | 1690.9 | 0.058 | 0.071 |
| 6 | 2.62 | 1475.2 | 1690.9 | 0.068 | 0.234 |
| 7 | 2.40 | 1475.5 | 1690.9 | 0.057 | 0.257 |
| 8 | 2.18 | 1476 | 1690.9 | 0.072 | 0.290 |
| 9 | 1.97 | 1476.2 | 1690.9 | 0.069 | 0.308 |
| 10 | 1.75 | 1472.2 | 1690.9 | 0.058 | 0.033 |
| 11 | 1.75 | 1474.5 | 1690.9 | 0.06 | 0.192 |
| 12 | 1.75 | 1473.7 | 1690.9 | 0.062 | 0.138 |
| 13 | 1.53 | 1474.4 | 1690.9 | 0.049 | 0.184 |
| 14 | 1.09 | 1472.7 | 1690.9 | 0.07 | 0.064 |
| 15 | 1.09 | 1472.1 | 1690.9 | 0.06 | 0.028 |
| 16 | 1.09 | 1471.6 | 1690.9 | 0.045 | -0.008 |
| 17 | 0.87 | 1472.1 | 1690.9 | 0.062 | 0.029 |
| 18 | 0.87 | 1472.5 | 1690.9 | 0.057 | 0.052 |
| 19 | 0.87 | 1472.4 | 1690.9 | 0.051 | 0.048 |
| 20 | 0.87 | 1471.9 | 1690.9 | 0.054 | 0.015 |
| 21 | 0.87 | 1472.6 | 1690.9 | 0.046 | 0.063 |
| 22 | 0.66 | 1472.1 | 1690.9 | 0.06 | 0.029 |
| 23 | 0.66 | 1471.9 | 1690.9 | 0.051 | 0.012 |
| 24 | 0.66 | 1472 | 1690.9 | 0.058 | 0.016 |
| 25 | 0.66 | 1472.2 | 1690.9 | 0.059 | 0.031 |
| 26 | 0.66 | 1472.2 | 1690.9 | 0.057 | 0.031 |
| 27 | 0.66 | 1471.3 | 1690.9 | 0.057 | -0.026 |
| 28 | 0.44 | 1471.5 | 1690.9 | 0.046 | -0.013 |
| 29 | 0.44 | 1471.9 | 1690.9 | 0.044 | 0.010 |
| 30 | 0.44 | 1471.8 | 1690.9 | 0.051 | 0.006 |
| 31 | 0.44 | 1471.8 | 1690.9 | 0.047 | 0.007 |
| 32 | 0.44 | 1471.9 | 1690.9 | 0.066 | 0.010 |
| 33 | 0.44 | 1471.8 | 1688.3 | 0.058 | 0.005 |
| 34 | 0.44 | 1471.8 | 1690.9 | 0.055 | 0.004 |
| 35 | 0.44 | 1471.9 | 1690.9 | 0.055 | 0.010 |
| 36 | 0.44 | 1471.7 | 1690.9 | 0.052 | -0.002 |
| 37 | 0.44 | 1471.9 | 1690.9 | 0.056 | 0.010 |
| 38 | 0.44 | 1471.9 | 1690.9 | 0.041 | 0.010 |
| 39 | 0.44 | 1471.9 | 1690.9 | 0.056 | 0.010 |
| 40 | 0.44 | 1471.9 | 1690.9 | 0.056 | 0.010 |
| 41 | 0.44 | 1471.7 | 1690.9 | 0.053 | 0.000 |
| 42 | 0.44 | 1471.7 | 1690.9 | 0.046 | 0.000 |
| 43 | 0.44 | 1471.9 | 1690.9 | 0.062 | 0.010 |
| 44 | 0.44 | 1471.7 | 1690.9 | 0.055 | 0.000 |
| 45 | 0.44 | 1471.7 | 1690.9 | 0.062 | 0.000 |
| 46 | 0.44 | 1471.9 | 1690.9 | 0.064 | 0.010 |
| 47 | 0.44 | 1471.9 | 1690.9 | 0.061 | 0.010 |

Fig. S1

a) Observed versus expected mean distribution of all alleles when considering all haplotypes.

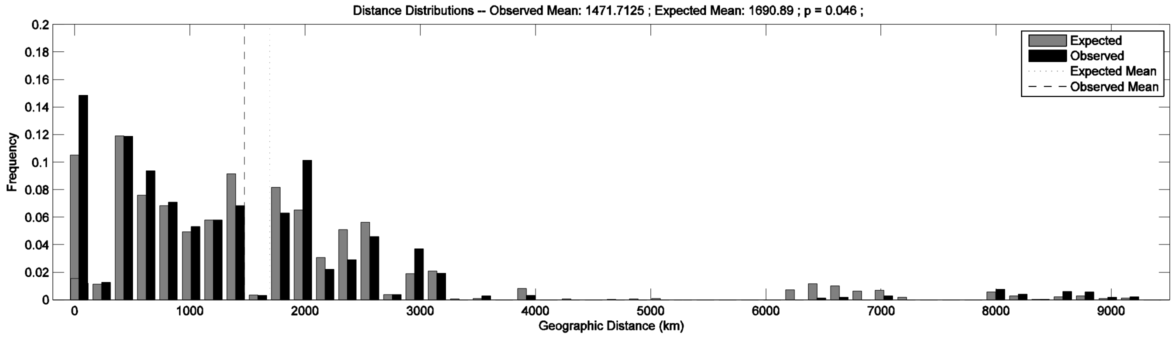


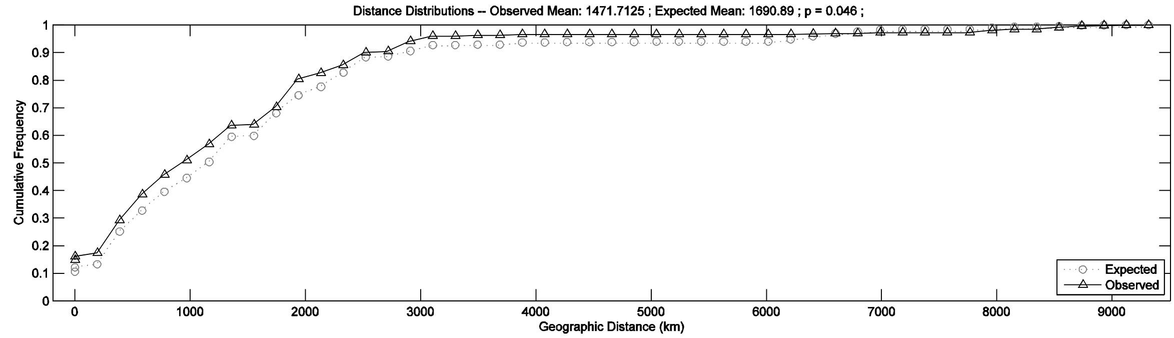


b) Haplotype-by-haplotype analysis of the complete C.ciconia dataset. The number of occurances of each haplotype (n) and significance level (p) are given along with the haplotypes specific SAShA statistics. The expected distribution is represented with circles and the observed by triangles.

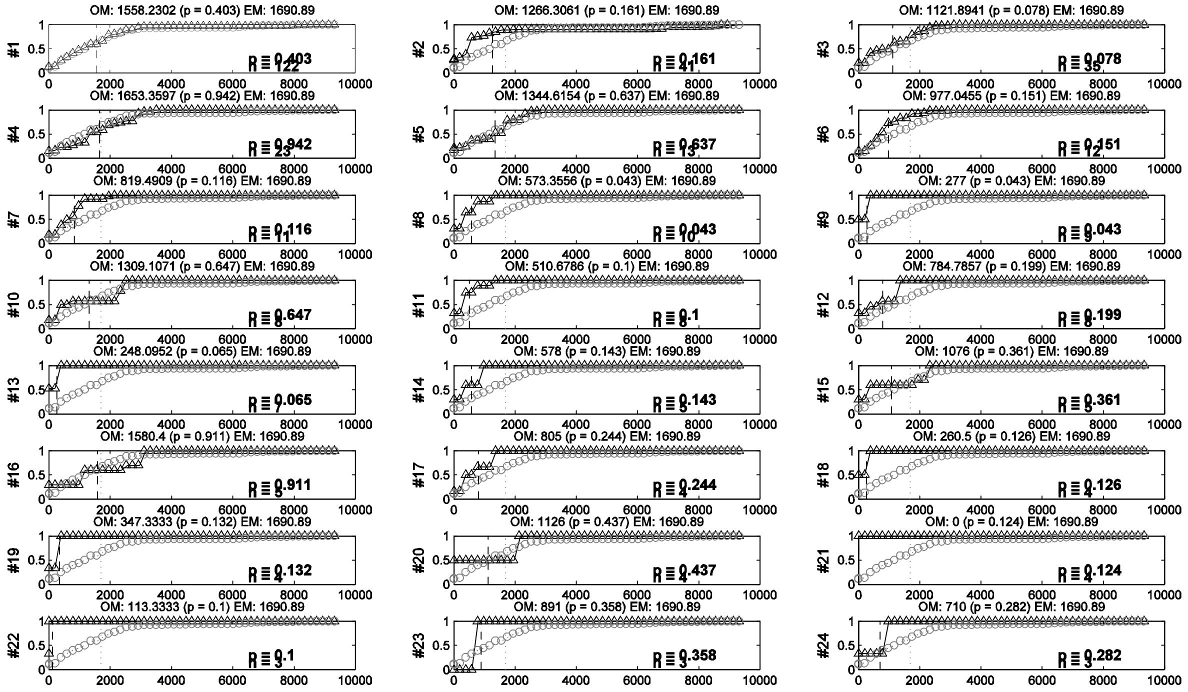

Fig. S2 Observed versus expected mean distribution of alleles with the most common haplotype (CC003) removed showing that the global distribution of CC003 overshadows some degree of underlying population structure.

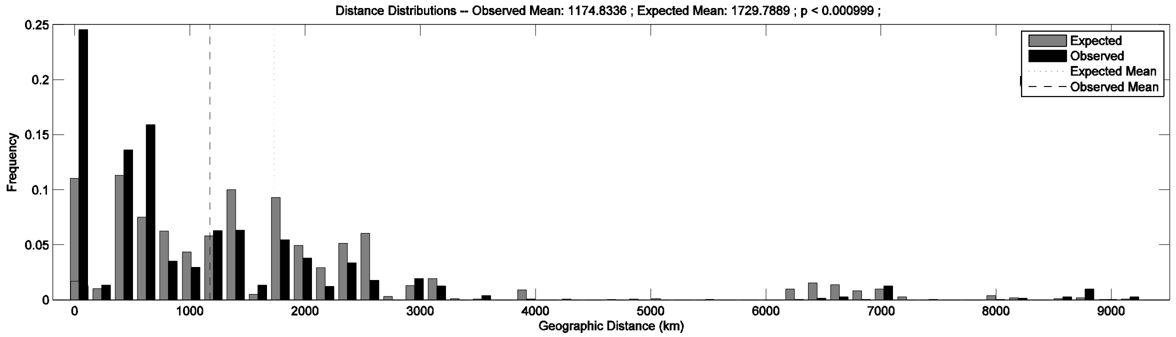


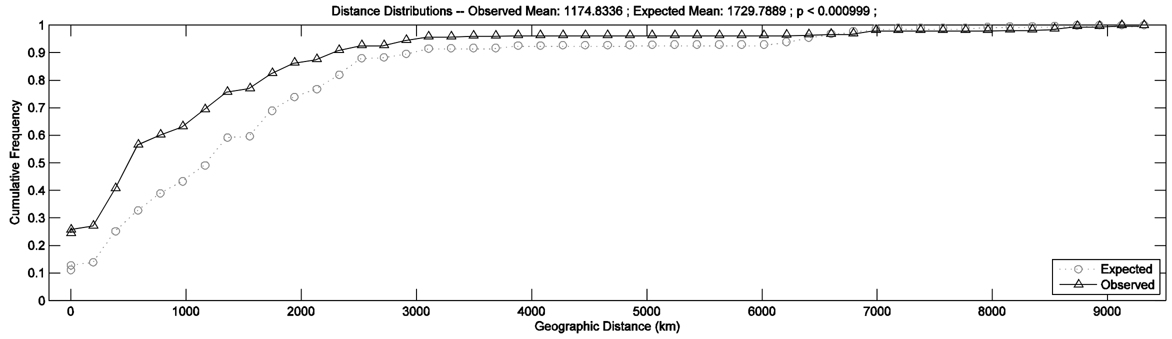

Supplement: Supplementary file 1 [file ece30003-4881-SD1.pdf]
