# Supplementary material for: Is population structure in the European white stork determined by flyway permeability rather than translocation history?
Source: Ecol Evol. 2013 Nov 7;3(15):4881–95. doi: 10.1002/ece3.845 (PMC3892355; doi:10.1002/ece3.845)

Supporting Information

Shephard J, Ogden R, Tryjanowski P, Olsson O, Galbusera P 201~~2~~3 ~~Unravelling the genetic effects of translocation history versus population decline on a widespread migrant: The case of the European white stork.~~

Is population structure in the European white stork determined by flyway permeability rather than translocation history?

Mitochondrial DNA Analysis

SAMOVA

To avoid artificially aggregating regions for analysis we used SAMOVA version 1.0 (Dupanloup *et al.* 2002), to define group structure. SAMOVA uses a spatial analysis of variance to maximise the variance among groups (F_{CT}) for a given value of k. Analyses were run for Historical and Contemporary samples independently and combined based on 100 initial conditions and 10000 steps for values of k = 2 to k = 10. Defined groups bore no resemblance to a geographic split among migration pools at any temporal scale. That is, defined groups were randomly comprised of regions from both sides of the migration divide. No other logical spatial partitioning of the data was found.

Microsatellite Analysis

2MOD

Deviation from migration/drift equilibrium was tested by comparing the relative likelihoods of 'geneflow/drift' and 'drift only' models with the program 2MOD, using a MCMC procedure with 10^5 iterations and a burn-in of 10^4 (Ciofi *et al.* 1999). Time intervals between population founding and population sampling were assumed sufficiently short such that

effects of mutations were negligible (drift only), while mutation rates were assumed much smaller than migration rates (geneflow/drift). Bayes factors were calculated to infer decisive power estimates of the most likely models (Goodman 1999; Jeffreys 1961). The results indicated equilibrium between gene flow and drift. Bayes factors were 1.4 (weak support) and 142 (very strong support) for Historical and Contemporary samples, respectively.

References

Ciofi C, Beaumont M, Swingland I, Bruford M (1999) Genetic divergence and units for conservation in the Komodo Dragon *Varanus komodoensis*. *Proceedings of the Royal Society of London Series B-Biological Sciences*, **266**, 2269-2274.

Dupanloup I, Schneider S, Excoffier L (2002) A simulated annealing approach to define the genetic structure of populations. *Molecular Ecology*, **11**, 2571-2581.

Goodman SN (1999) Toward Evidence-Based Medical Statistics. 2: The Bayes Factor. *Annals of Internal Medicine*, **130**, 1005-1013.

Jeffreys H (1961) *Theory of probability third edition*, Third edn. Oxford University Press, Oxford.

Supporting Tables

Table S1 Museum sample information and museum accession codes. Sequence labels and mtDNA haplotype designations refer to the current study (*n* = 54).

Table S2 Variable sites among unique haplotypes for Ciconia ciconia across *n* = 373bp of control region.

Table S3 Count of the regional distribution of unique haplotypes and their Genbank Accession Numbers. Haplotype codes in bold were found in the Historical sample set. CC011 and CC016, marked with an asterix, were the only haplotypes found in both the Historical and Contemporary dataset

Table S4 SAShA jackknife results for all alleles including the most common (CC03) seen here as Allele 1. Alleles correspond to haplotypes ranked according to frequency. Each row represents an analysis with each allele removed sequentially. The test statistic OM, describes the observed mean distance between alleles. Where OM is less than the expected mean (EM), alleles are considered to be aggregated (under-distributed). Where OM is larger than EM, alleles are considered overdispersed (panmictic). A jackknifing procedure allows the identification of which alleles contribute to the distribution.

Supporting Figures

Fig. S1 a) Observed (OM) versus expected mean (EM) distribution of alleles when considering all haplotypes. b) Haplotype-by-haplotype analysis of the complete *C.ciconia* dataset. The number of occurances of each haplotype (*n*) and significance level (*p*) are given along with the haplotype specific SAShA statistics. The expected distribution is represented with circles and the observed by triangles. Allele 8 (CC040) and Allele 9 (CC005) show significant aggregation. Geographic distance (km) and Cumulative frequency of alleles describe the x and y axis respectively.

Fig. S2 Observed versus expected mean distribution of alleles with the most common haplotype (CC003) removed showing that the global distribution of CC003 overshadows some degree of underlying population structure.

Table S1 Museum sample information and museum accession codes. Sequence labels and mtDNA haplotype designations refer to the current study (N=54)

Species	Country of Holding	Location of Holding	Source Location	Birth Country	Suggested Mig Pool	Accession No.	Accession Year	Latitude DD	Longitude DD	Sample Type	Sequence Label	mtDNA Haplotype	GenBank Accession No
Ciconia ciconia	Belgium	Brussels Natural History Museum	Africa,Belgian Congo-Nat Park Upenba	Africa	East	BNHM55752	1946	-9.1999	25.8500	Dried Toe Pad	AFR_O_01	CC056	JN411006
Ciconia ciconia	Belgium	Brussels Natural History Museum	Africa,Belgian Congo-Nat Park Nawangolu Uele	Africa	East	BNHM22.577	1948	-9.1999	25.8500	Dried Toe Pad	AFR_O_02	CC056	JN411006
Ciconia ciconia	Belgium	Brussels Natural History Museum	Africa, Belgian Congo-Tanganika (Musosa)	Africa	East	BNHM20578	1941	-9.1999	25.8500	Dried Toe Pad	AFR_O_03	CC057	JN411007
Ciconia ciconia	Belgium	Brussels Natural History Museum	Africa, Belgian Congo-Tanganika (Musosa)	Africa	East	BNHM20577	1942	-9.1999	25.8500	Dried Toe Pad	AFR_O_04	CC058	JN411008
Ciconia ciconia	Belgium	Brussels Natural History Museum	Belgium, Knokke	Belgium	West	BNHM449256	1931	51.3500	3.2667	Dried Toe Pad	BE_O_223	CC010	JN410960
Ciconia ciconia	Belgium	Brussels Natural History Museum	Belgium, Liege	Belgium	West	BNHM449257	?	50.6333	5.5667	Dried Toe Pad	BE_O_224	CC011	JN410961
Ciconia ciconia	Belgium	Brussels Natural History Museum	Belgium	Belgium	West	BNHM449258	1932	50.8333	4.3333	Dried Toe Pad	BE_O_225	CC012	JN410962
Ciconia ciconia	Belgium	Brussels Natural History Museum	Belgium, Alsemberg	Belgium	West	BNHM451409	1935	50.7333	4.3167	Dried Toe Pad	BE_O_226	CC013	JN410963
Ciconia ciconia	Belgium	Brussels Natural History Museum	Belgium, Alsemberg	Belgium	West	BNHM451410	1935	50.7333	4.3167	Dried Toe Pad	BE_O_227	CC014	JN410964
Ciconia ciconia	Belgium	Brussels Natural History Museum	Belgium, Saint Gerard	Belgium	West	BNHM451456	?	50.3500	4.7500	Dried Toe Pad	BE_O_228	CC015	JN410965
Ciconia ciconia	Belgium	Brussels Natural History Museum	Belgium, Brussels	Belgium	West	BNHM451792	1829	50.8333	4.3333	Dried Toe Pad	BE_O_229	CC016	JN410966
Ciconia ciconia	Belgium	Brussels Natural History Museum	France, Cerfontaine	France	West	BNHM453414	1946	50.2667	4.0333	Dried Toe Pad	FR_O_056	CC011	JN410961
Ciconia ciconia	Belgium	Brussels Natural History Museum	Lüneburg, Germany	Germany	East	Helgoland208011	1931	53.3667	10.4833	Dried Toe Pad	GR_022	CC106	JN411056
Ciconia ciconia	Belgium	Brussels Natural History Museum	Russia, Rositten	Russia	East	BNHM450894	1935	50.4000	20.4333	Dried Toe Pad	POL_O_64	CC031	JN410981
Ciconia ciconia	Germany	Museum für Naturkunde, Berlin	Tansania	Africa	East	2000/15789	1895-1910	-6.3688	34.8888	Dried Toe Pad	AFR_O_05	CC058	JN411008
Ciconia ciconia	Germany	Museum für Naturkunde, Berlin	Tansania	Africa	East	2000/15791	1909	-6.3688	34.8888	Dried Toe Pad	AFR_O_06	CC059	JN411009
Ciconia ciconia	Germany	Museum für Naturkunde, Berlin	Ufipa, Tanzania	Africa	East	2000/15793	1909	-6.3688	34.8888	Dried Toe Pad	AFR_O_07	CC060	JN411010
Ciconia ciconia	Germany	Museum für Naturkunde, Berlin	Ufipa, Tanzania	Africa	East	2000/15794	1909	-6.3688	34.8888	Dried Toe Pad	AFR_O_08	CC058	JN411008
Ciconia ciconia	Germany	Museum für Naturkunde, Berlin	Ufipa, Tanzania	Africa	East	2000/15796	1909	-6.3688	34.8888	Dried Toe Pad	AFR_O_09	CC011	JN410961
Ciconia ciconia	Germany	Museum für Naturkunde, Berlin	Tansania	Africa	East	2000/15797	1903	-6.3688	34.8888	Dried Toe Pad	AFR_O_10	CC061	JN411011
Ciconia ciconia	Germany	Museum für Naturkunde, Berlin	Tansania	Africa	East	2000/15798	1903	-6.3688	34.8888	Dried Toe Pad	AFR_O_11	CC062	JN411012
Ciconia ciconia	Germany	Museum für Naturkunde, Berlin	Hellenurme	Estonia	East	2000/15790	1895	58.1361	26.3899994	Dried Toe Pad	EST_O_1	CC031	JN410981
Ciconia ciconia	Germany	Museum für Naturkunde, Berlin	Malchow	Germany	West/East	1990/9	Unk	53.4667	12.4167	Dried Toe Pad	GR_017	CC068	JN411018
Ciconia ciconia	Germany	Museum für Naturkunde, Berlin	Miletkova	Macedonia	East	2000/15795	1917	41.6074	21.7449	Dried Toe Pad	MAC_O_001	CC011	JN410961
Ciconia ciconia	Netherlands	Naturalis - Natural History Museum, Leiden	Beja, Tunisia	Africa	West	NNHM19574	1951	36.7333	9.1833	Dried Toe Pad	AFR_O_12	CC063	JN411013
Ciconia ciconia	Netherlands	Naturalis - Natural History Museum, Leiden	s Hertongenbosch	Netherlands	West	NNHM545/CAT13	1906	51.7000	5.3167	Dried Toe Pad	NL_O_05	CC011	JN410961
Ciconia ciconia	Netherlands	Naturalis - Natural History Museum, Leiden	s Hertongenbosch	Netherlands	West	NNHM545/CAT14	1906	51.7000	5.3167	Dried Toe Pad	NL_O_06	CC011	JN410961
Ciconia ciconia	Netherlands	Naturalis - Natural History Museum, Leiden	Slikkerveer	Netherlands	West	NNHM4875	?	51.8833	4.6167	Dried Toe Pad	NL_O_07	CC011	JN410961
Ciconia ciconia	Netherlands	Naturalis - Natural History Museum, Leiden	Anholt	Netherlands	West	NNHM7103	1910	52.7833	6.4167	Dried Toe Pad	NL_O_08	CC011	JN410961
Ciconia ciconia	Netherlands	Naturalis - Natural History Museum, Leiden	Assen	Netherlands	West	NNHM9141	1939	53.0000	6.5500	Dried Toe Pad	NL_O_09	CC017	JN410967
Ciconia ciconia	Netherlands	Naturalis - Natural History Museum, Leiden	Huizum	Netherlands	West	NNHM13340	1947	53.2000	5.8000	Dried Toe Pad	NL_O_10	CC012	JN410962
Ciconia ciconia	Netherlands	Naturalis - Natural History Museum, Leiden	Veenwouden	Netherlands	West	NNHM15356	1950	53.2333	6.0000	Dried Toe Pad	NL_O_11	CC011	JN410961
Ciconia ciconia	Netherlands	Naturalis - Natural History Museum, Leiden	Driebergen	Netherlands	West	NNHMCAT9	1865	52.0500	5.2833	Dried Toe Pad	NL_O_12	CC011	JN410961
Ciconia ciconia	Netherlands	Naturalis - Natural History Museum, Leiden	Ausgustinusga	Netherlands	West	NNHM24306	1956	53.2167	6.1667	Dried Toe Pad	NL_O_13	CC011	JN410961
Ciconia ciconia	Netherlands	Naturalis - Natural History Museum, Leiden	Wesermundung	Netherlands	West	NNHM24703	1891	53.2833	5.5667	Dried Toe Pad	NL_O_14	CC011	JN410961
Ciconia ciconia	Netherlands	Naturalis - Natural History Museum, Leiden	Drachten	Netherlands	West	NNHM15344	1950	53.1000	6.1000	Dried Toe Pad	NL_O_15	CC011	JN410961
Ciconia ciconia	Netherlands	Naturalis - Natural History Museum, Leiden	Schoonhoven	Netherlands	West	NNHM25017	1957	51.9500	4.8500	Dried Toe Pad	NL_O_16	CC011	JN410961
Ciconia ciconia	Netherlands	Naturalis - Natural History Museum, Leiden	Papendrecht	Netherlands	West	NNHM43102	1959	51.8333	4.6833	Dried Toe Pad	NL_O_17	CC011	JN410961
Ciconia ciconia	Netherlands	Naturalis - Natural History Museum, Leiden	Oegstgeest	Netherlands	West	NNHMCAT8	1865	52.1833	4.4667	Dried Toe Pad	NL_O_18	CC012	JN410962
Ciconia ciconia	Netherlands	Naturalis - Natural History Museum, Leiden	Sassenheim	Netherlands	West	NNHMCAT3	1859	52.2167	4.5333	Dried Toe Pad	NL_O_19	CC011	JN410961
Ciconia ciconia	Netherlands	Naturalis - Natural History Museum, Leiden	South Holland	Netherlands	West	NNHMCAT209-1	1851	52.1500	4.5000	Dried Toe Pad	NL_O_20	CC011	JN410961
Ciconia ciconia	Netherlands	Naturalis - Natural History Museum, Leiden	Leiden	Netherlands	West	NNHMCAT209-2	1866	52.1500	4.5000	Dried Toe Pad	NL_O_21	CC018	JN410968
Ciconia ciconia	Netherlands	Naturalis - Natural History Museum, Leiden	Latakoe	South Africa	East	NNHMCAT16	1834	-33.9167	18.4167	Dried Toe Pad	SAF_O_01	CC011	JN410961
Ciconia ciconia	Sweden	Museum Zoology, Lund University	Råby, Lund	Sweden	East	L846/30313	1846	55.6854	13.2084	Dried Toe Pad	SW_SW_O_14	CC011	JN410961
Ciconia ciconia	Sweden	Museum Zoology, Lund University	Unknown	Sweden	East	L848/6133	1848	?	?	Dried Toe Pad	SW_SW_O_15	CC011	JN410961
Ciconia ciconia	Sweden	Museum Zoology, Lund University	Trollenäs	Sweden	East	L871/3009	1871	55.8659	13.2444	Dried Toe Pad	SW_SW_O_16	CC016	JN410966
Ciconia ciconia	Sweden	Museum Zoology, Lund University	Fjelie	Sweden	East	L872/3011	1872	55.7273	13.1047	Dried Toe Pad	SW_SW_O_17	CC034	JN410984
Ciconia ciconia	Sweden	Museum Zoology, Lund University	Bergsjöholm	Sweden	East	L876/3038	1876	55.4513	13.7773	Dried Toe Pad	SW_SW_O_18	CC017	JN410967
Ciconia ciconia	Sweden	Museum Zoology, Lund University	Stehag	Sweden	East	L877/3051	1877	55.9015	13.4004	Dried Toe Pad	SW_SW_O_19	CC035	JN410985
Ciconia ciconia	Sweden	Museum Zoology, Lund University	Skabersjö	Sweden	East	L879/3018	1879	55.5401	13.1496	Dried Toe Pad	SW_SW_O_20	CC011	JN410961
Ciconia ciconia	Sweden	Museum Zoology, Lund University	Farhult	Sweden	East	L909/3500	1909	56.2141	12.7124	Dried Toe Pad	SW_SW_O_21	CC011	JN410961
Ciconia ciconia	Sweden	Museum Zoology, Lund University	Skårby	Sweden	East	L925/3032	1925	55.4866	13.7132	Dried Toe Pad	SW_SW_O_22	CC011	JN410961
Ciconia ciconia	Sweden	Museum Zoology, Lund University	Silvåkra	Sweden	East	L948/3072	1948	55.6844	13.4944	Dried Toe Pad	SW_SW_O_23	CC036	JN410986
Ciconia ciconia	Sweden	Museum Zoology, Lund University	Hörja	Sweden	East	L954/3013	1954	56.2056	13.5898	Dried Toe Pad	SW_SW_O_24	CC011	JN410961

NB. Sample BNHM450894 was sampled in Russia, but the Lat/Long places it in Poland. This may be due to geographic boundary changes associated with the World war. It was included in the Polish sample for analysis purposed. Irrespective of this it is considered to belong to the eastern migration pool so allocation to sample group is irrelevant at this scale

Table S2 – Variable sites among unique Haplotypes for *Ciconia ciconia* across *n* = 373bp of control region.

			11111111	1111111111	1111111111	1111111111	2222222222	2222233333	33333
	3345555566	6777778889	9900011111	1122333333	4444455555	6677789999	0111112244	5666600011	12333
	5813678925	7013572570	7903801236	7903023468	0268914589	1915642569	7015781356	6015812545	82034
Concensus	CGTTTACTTC	-ACTATCAAA	GGCAAGCGTA	TAAT-TTTGC	GAAGTGTTGC	TGGCTTAGGC	GGGAAATCAA	GGGTGGATAA	GAGAC
CC001	....	-........G	..T.......	...C-.....	....	....	........G.	.......C..	.....
CC002	....	-........G	....	....A.....	.....A....	....	....	.......C..	.....
CC003	....	-.........	....	....-.....	.........T	....	...G......	....	.....
CC004	....	-........G	....	.G..-.....	....	....	....	....	.....
CC005	....	-........G	....	....-.....	.....A....	....	....	.......C..	.....
CC006	....	-........G	..T.......	....-.....	....	....	........G.	....	.....
CC007	....	-........G	....	....-.....	....	.......A..	....	....	.....
CC008	....	-.........	....	....-.....	.........T	....	...G......	.......C..	.....
CC009	....	-........G	....	....-.....	....	....	........G.	....	.....
CC010	....	-G........	..T.......	....-.....	.........T	....	....	....	.....
CC011	....	-.........	..T.......	....-.....	.........T	....	...G......	....	.....
CC012	....	-.........	..T.......	....-....T	.........T	....	...G......	....	.....
CC013	....	-.........	..T.......	....-.....	.........T	....C.....	...G......	.....A....	.....
CC014	....	-.........	..T.......	....-.....	.........T	....C.....	...G......	....	.....
CC015	....	-.........	..T.......	....-.....	...A.....T	....	...G......	.AA.......	.....
CC016	....	-.........	..T.......	....-.....	.........T	....	....	....	.....
CC017	....	-........G	..T.......	....-.....	....	.......A..	....	....	.....
CC018	....	-.........	..T.......	....-.....	.........T	....	...G......	....	A....
CC019	....	-........G	....	....-.....	....	....	....	....	.....
CC020	....	-.........	....	....-.....	.........T	....	....	....	.....
CC021	.AC.AGTGGT	A.TATCACT.	TATTCTTAC.	C.GCG.ACAG	C.T.A-GA..	GT-A.AG...	....	........G.	....T
CC022	....	-........G	....	....-.....	....	....	....	.......C..	.....
CC023	....	-.........	....	....-.....	....	....	...G......	....	.....
CC024	....	-........G	....	.G..-.....	....	....	....	...C......	.....
CC025	....	-.........	....	....-.....	.........T	....	....	.......C..	.....
CC026	....	-.........	.........G	....-.....	.........T	....	...G......	....	.....
CC027	....	-........G	....	....-.....	.........T	....	...G......	....	.....
CC028	.AC.AGTGGT	A.TATCACT.	TATTCTTAC.	C.GCG.ACAG	C.T.A-GA..	GT-A.AG...	.........G	.......C..	.....
CC029	....	-.........	....	....-C....	.........T	....	...G......	....	.....
CC030	....	-.........	....	....-.....	.........T	......G...	...G......	....	.....

```
CC031       .......... -......... ..T....... ....-..... .........T .......... ...G...... .....A.... .....
CC032       .......... -........G .......... ...C-..... .........  .......... .......... .........G .....
CC033       .......... -......... .......... ....-..... .........T .A........ ...G...... .......... .....
CC034       .......... -........G ..T....... .G..-..... .........  .......... .......... ...C...... .....
CC035       .......... -......... ..T....... ....-..... .........T .......... ...G...... .......C.. .....
CC036       .......... -......... ..T....... ....-..... .........T .........T ...G...... .......... .....
CC037       .......... -......... .......... ....A..... .........T .......... ...G...... .......... .....
CC038       .......... -......... .......... ....-..... .........T ......G... A..G...... .......... .....
CC039       .......... -......... .......... ....-..... .G.......T .......... ..AG..C... .......... .....
CC040       .......... -......... .......... ...C-..... .........T .......... .......... .......... .....
CC041       .......... -......... ..T....... ...C-..... .........T .......... .......... .......... .....
CC042       .AC.AGTGGT A.TATCACT. TATTCTTAC. C.GCG.ACAG C.T.A-GA.. GT-A.AG... .......... .......... .....
CC043       .......... -........G ..T....... .G..-..... .......... ......G... .......... .......... .....
CC044       .......... -........G .......... .G..-..... .......... ......G... .......... .......... .....
CC045       .AC.AGTGGT A.TATCACT. TATTCTTAC. C.GCG.ACAG C.T.A-GA.. GT-A.A.... .......... .......... .....
CC046       .......... -......... .......... ....-..... .........T .....C.... ...G...... .......... ....T
CC047       .......... -........G .......... .G..-..... .......... .......... .......... .......... ....T
CC048       .......... -........G ..T....... ....-..... .......... .......... .....G.T.. .......... ....T
CC049       .......... -........G .......... .G..-..... .......... .A........ ...G...... .......... .....
CC050       .AC.AGTGGT A.TATCACT. TATTCTTAC. C.GCG.ACAG C.T.A-GA.. GT-A.AG... ...G...... .......... ....T
CC051       .......... -......... .......... ....-..... .........T .....C.... ...G...... .......... .....
CC052       .AC.AGTGGT A.TATCACT. TATTCTTAC. C.GCG.ACAG C.T.A-GA.. GT-A.A.... .......... .......... .G..T
CC053       .AC.AGTGGT A.TATCACT. TATTCTTAC. C.GCG.ACAG C.T.A-GA.. GT-A.AG... .......... .......... ...G.
CC054       .......... -........G .......... .G..-..... .......... .......... .......... .......... .G..T
CC055       .......... -......... ..T....... ....-..... .........T .A........ ...G...... .......... .....
CC056       .......... -......... ..T....... ....-..... .........T .......... ...G...... .........G .....
CC057       .......... -........G ..T....... ...C-..... .......... .......... .......... ........G. .....
CC058       .......... -........G ..T        ....-..... .......... .......... .......... .......... .....
CC059       .......... -........G ..T....... ....-..... .......... .......... .......... .......C.. .....
CC060       .......... -......... ..T....... ...C-C.... .........T .......... ...G...... .......... A....
CC061       .......... -........G ..T....... ....-..... .......... ......GA.. .......... .......... .....
CC062       .......... -......... ..T....... ....-..... .........T .......... ...G...... .......... ....T
CC063       .......... -........G ..T....... .G..-..... .......... .......... ........G. ...C...... .....
CC064       .......... -......... .......... ....-..... .........T ..A....... ...G...... .......... .....
CC065       .......... -......... .......... ....-...A. .........T .......... ...G...... .......... .....
CC066       .......... -........G .......... ....-..... .......... ......GA.. .......... .......... .....
CC067       .......... -........G .......T.. .G..-..... .....T.... .......... .A........ ...C...... .....
CC068       .AC.AGTGGT A.TATCACT. TATTCTTAC. C.GCG.ACAG C.T.A-GA.. GT-A.AG... ...G...... .......... .....
```

```
CC069    .......... -......... .......... ....-..... .........T ........A. ...G...... .......... .....
CC070    .AC.AGTGGT A.TATCACT. TATTCTTAC. C.GCG.ACAG C.T.A-GA.. GT-A.A.... .......... .......... ....T
CC071    .......... -......... .......... ....-..... .........T .......... .......T.. .......... .....
CC072    .......... -........G .......... ....-..... .......... .A........ .........G .......... .....
CC073    .AC.AGTGGT A.TATCACT. TATTCTTAC. C.GCG.ACAG C.T.A-GA.. GT-A.AG... .......... .......C.. .....
CC074    .......... -........G .......... .G..-..... .......... .......... .......... A......... .....
CC075    .......... -........G .......... .G..-..... .......... .......... .......... ........G. .....
CC076    .......... -......... .......... ....-..... .G.......T .......... ...G..C... .......... .....
CC077    .......... -........G .......... ....-..... .......... .......... .........G .......... .....
CC078    .......... -........G .......... .G..-..... .......... ....C..... .......... .......... .....
CC079    .AC.AGTGGT A.TATCACT. TATTCTTAC. C.GCG.ACAG C.T.A-GA.. GT-A.AG... .......... .......C.. ..A..
CC080    .......... -........G .......... ....-..... .......... .......... ......C... .......C.. .....
CC081    .ACCAGTGGT A.TATCACT. TATTCTTAC. C.GCG.ACAG C.T.A-GA.. GT-A.AG... .......T.. .......... .....
CC082    TAC.AGTGGT A.TATCACT. TATTCTTAC. C.GCG.ACAG C.T.A-GAA. GT-A.AG... .......... .......... .....
CC083    .......... -......... .......... ...C-..... .........T .A........ .......... .......... .....
CC084    .......... -........G .......... .G..-..... .......... .......... .......T.. .......... .....
CC085    .AC.AGTGGT A.TATCACT. TATTCTTAC. C.GCG.ACAG C.T.A-GA.. GT-A.AG... .......... .........G .....
CC086    .......... -......... .......... ....-..... .........T .......... ...G...... ....A..... .....
CC087    .......... -........G .......... ....-..... .....T.... .......... ......C... .......C.. .....
CC088    .......... -........G .......... .G..-..... .......... .......... ....G..... .......... ....T
CC089    .......... -........G .......... .G..-..... .......... .A........ .......... .......... .....
CC090    .......... -........G .......... ....-..... .......... .A........ .......... .......... .....
CC091    .......... -........G ..T....... ....-..... .....T.... .......... ........G. .......... .....
CC092    .AC.AGTGGT A.TATCACT. TATTCTTAC. C.GCG.ACAG C.T.A-GA.. GT-A.AG... .......... ...C...... .....
CC093    .......... -......... .......... ....-..... .....T...T .......... .......... .......... .....
CC094    .......... G-........ .......... ....-..... .........T .......... .......... .......... A....
CC095    .AC.AGTGGT A.TATCACT. TATTCTTAC. C.GCG.ACAG C.T.A-GA.. GT-A.AG... .........G .......... .....
CC096    .......... -......... .......... ...C-..... .........T ......G... A..G...... .......... .....
CC097    .......... -......... .......... ....-....T .........T .......... ...G...... .......... .....
CC098    .......... -........G .......... .G..-..... .......... .......... .......... ......G... .....
CC099    .........T -........G .......... .G..-..... .......... .......... .......... .......... .G..T
CC100    .......... -......G.. .......T.. ....-..... .....T...T .......... ...G...... .......... .....
CC101    .......... -......... .......... ....-C.... .........T .......... .......... .......... ....T
CC102    .......... -........G .......... .G..-..... .......... ......G... .......... ...C...... .....
CC103    .AC.AGTGGT A.TATCACT. TATTCTTAC. C.GCG.ACAG C.T.A-GA.. GT-A.AG... ...G...... .......... A....
CC104    .......... -........G .......... .G..-..... .......... .......... .......... ......G... A....
CC105    .......... -......... .......... ....-..... .........T .........T ...G...... .......... .....
CC106    .......... -........G ..T....... ...C-..... .......... .......... .......... .......... .....
```

Table S3 Regional distribution of haplotypes. Haplotype codes in bold were found in the Historical sample set. CC011 and CC016 marked with an asterix, were the only haplotypes found in both the Historical and Contemporary dataset.

MtDNA Haplotype	Genbank Accession No.	Africa_a	Africa_b	Africa_c	Algeria	Austria	Belgium	Czech Republic	Estonia	France	Germany	Latvia	Macedonia	Netherlands	Poland	Portugal	Slovakia	South Africa	Spain	Sweden	Israel	Unknown	Total
CC001	NC_002197																					1	1
CC002	JN410952						1																1
CC003	JN410953				6	3	9	1		12	8	3		7	25	7	3	2	28	4	4		122
CC004	JN410954						7			1		1			4	11	1		10				35
CC005	JN410955						6			3													9
CC006	JN410956						1														1		2
CC007	JN410957				1		2			1				2	2	5							13
CC008	JN410958						2			4					3		1		1				11
CC009	JN410959						2																2
**CC010**	JN410960						1																1
**CC011***	JN410961		1				1			2	1		1	20	6		1	1	1	6			41
**CC012**	JN410962						1							2									3
**CC013**	JN410963						1																1
**CC014**	JN410964						1																1
**CC015**	JN410965						1																1
**CC016***	JN410966					1	1													1			3
**CC017**	JN410967													1						1			2
**CC018**	JN410968													1									1
CC019	JN410969					1	1			4		1		1	3	1							12
CC020	JN410970				8					2	3	1			2	3			3	1			23
CC021	JN410971														1		1						2
CC022	JN410972									1	1				3								5
CC023	JN410973														1								1
CC024	JN410974														3	2	2			1			8
CC025	JN410975														2								2
CC026	JN410976														1								1
CC027	JN410977														1								1
CC028	JN410978														1								1
CC029	JN410979														1	3			1				5
CC030	JN410980														1								1
**CC031**	JN410981														1								1
CC032	JN410982									1										3	1		5
CC033	JN410983																			1			1
**CC034**	JN410984																			1			1
**CC035**	JN410985								1											1			2
**CC036**	JN410986																			1			1
CC037	JN410987																			1			1
CC038	JN410988																			1			1
CC039	JN410989																		1				1
CC040	JN410990									2						3			5				10
CC041	JN410991									1									1				2
CC042	JN410992									1						2			1				4
CC043	JN410993																		1				1
CC044	JN410994									1						3			4				8
CC045	JN410995																		1				1
CC046	JN410996															1			3				4
CC047	JN410997																		1				1
CC048	JN410998															2			2				4
CC049	JN410999															5			2				7
CC050	JN411000									3						4			1				8
CC051	JN411001																		1				1
CC052	JN411002																		2				2
CC053	JN411003									1		1											2
CC054	JN411004					1																	1
CC055	JN411005					1																	1
**CC056**	JN411006	2																					2
**CC057**	JN411007	1																					1
**CC058**	JN411008	1	2																				3
**CC059**	JN411009		1																				1
**CC060**	JN411010		1																				1
**CC061**	JN411011		1																				1
**CC062**	JN411012		1																				1
**CC063**	JN411013			1																			1
CC064	JN411014																	1					1
CC065	JN411015							3															3
CC066	JN411016										3					1							4
CC067	JN411017										1												1
CC068	JN411018										1												1
CC069	JN411019										1												1
CC070	JN411020															1							1
CC071	JN411021															2							2
CC072	JN411022															2							2
CC073	JN411023															2							2
CC074	JN411024															1							1
CC075	JN411025															1							1
CC076	JN411026															4							4
CC077	JN411027									1						1							2
CC078	JN411028									1						1							2
CC079	JN411029															1							1
CC080	JN411030															3							3
CC081	JN411031															1					2		3
CC082	JN411032															2							2
CC083	JN411033															1							1
CC084	JN411034															1							1
CC085	JN411035									1						1							2
CC086	JN411036									1						1							2
CC087	JN411037									1													1
CC088	JN411038									1													1
CC089	JN411039									1													1
CC090	JN411040									1													1
CC091	JN411041									1													1
CC092	JN411042									1													1
CC093	JN411043									1													1
CC094	JN411044									2													2
CC095	JN411045									2													2
CC096	JN411046									1													1
CC097	JN411047									1													1
CC098	JN411048																1						1
CC099	JN411049																1						1
CC100	JN411050																1						1
CC101	JN411051													1									1
CC102	JN411052						1																1
CC103	JN411053																				1		1
CC104	JN411054																				1		1
CC105	JN411055																				1		1
**CC106**	JN411056										1												1
		4	7	1	15	7	39	4	1	57	20	7	1	35	61	79	12	4	70	23	11		459

Table S4 SAShA jackknife results for all alleles including the most common (CC03) seen here as Allele 1.

Allele jacknifed	% of Data	OM	EM	*P*	% Change in OM
0	-	1471.7	1690.9	0.046	0.000
**1**	**26.64**	**1174.8**	**1690.9**	**0**	**-20.172**
2	8.95	1491	1690.1	0.11	1.314
3	7.64	1495	1690.9	0.13	1.583
4	5.02	1466.8	1690.9	0.055	-0.337
5	2.84	1472.8	1690.9	0.058	0.071
6	2.62	1475.2	1690.9	0.068	0.234
7	2.40	1475.5	1690.9	0.057	0.257
8	2.18	1476	1690.9	0.072	0.290
9	1.97	1476.2	1690.9	0.069	0.308
10	1.75	1472.2	1690.9	0.058	0.033
11	1.75	1474.5	1690.9	0.06	0.192
12	1.75	1473.7	1690.9	0.062	0.138
13	1.53	1474.4	1690.9	0.049	0.184
14	1.09	1472.7	1690.9	0.07	0.064
15	1.09	1472.1	1690.9	0.06	0.028
16	1.09	1471.6	1690.9	0.045	-0.008
17	0.87	1472.1	1690.9	0.062	0.029
18	0.87	1472.5	1690.9	0.057	0.052
19	0.87	1472.4	1690.9	0.051	0.048
20	0.87	1471.9	1690.9	0.054	0.015
21	0.87	1472.6	1690.9	0.046	0.063
22	0.66	1472.1	1690.9	0.06	0.029
23	0.66	1471.9	1690.9	0.051	0.012
24	0.66	1472	1690.9	0.058	0.016
25	0.66	1472.2	1690.9	0.059	0.031
26	0.66	1472.2	1690.9	0.057	0.031
27	0.66	1471.3	1690.9	0.057	-0.026
28	0.44	1471.5	1690.9	0.046	-0.013
29	0.44	1471.9	1690.9	0.044	0.010
30	0.44	1471.8	1690.9	0.051	0.006
31	0.44	1471.8	1690.9	0.047	0.007
32	0.44	1471.9	1690.9	0.066	0.010
33	0.44	1471.8	1688.3	0.058	0.005
34	0.44	1471.8	1690.9	0.055	0.004
35	0.44	1471.9	1690.9	0.055	0.010
36	0.44	1471.7	1690.9	0.052	-0.002
37	0.44	1471.9	1690.9	0.056	0.010
38	0.44	1471.9	1690.9	0.041	0.010
39	0.44	1471.9	1690.9	0.056	0.010
40	0.44	1471.9	1690.9	0.056	0.010
41	0.44	1471.7	1690.9	0.053	0.000
42	0.44	1471.7	1690.9	0.046	0.000
43	0.44	1471.9	1690.9	0.062	0.010
44	0.44	1471.7	1690.9	0.055	0.000
45	0.44	1471.7	1690.9	0.062	0.000
46	0.44	1471.9	1690.9	0.064	0.010
47	0.44	1471.9	1690.9	0.061	0.010

Fig. S1

a) Observed versus expected mean distribution of all alleles when considering all haplotypes.

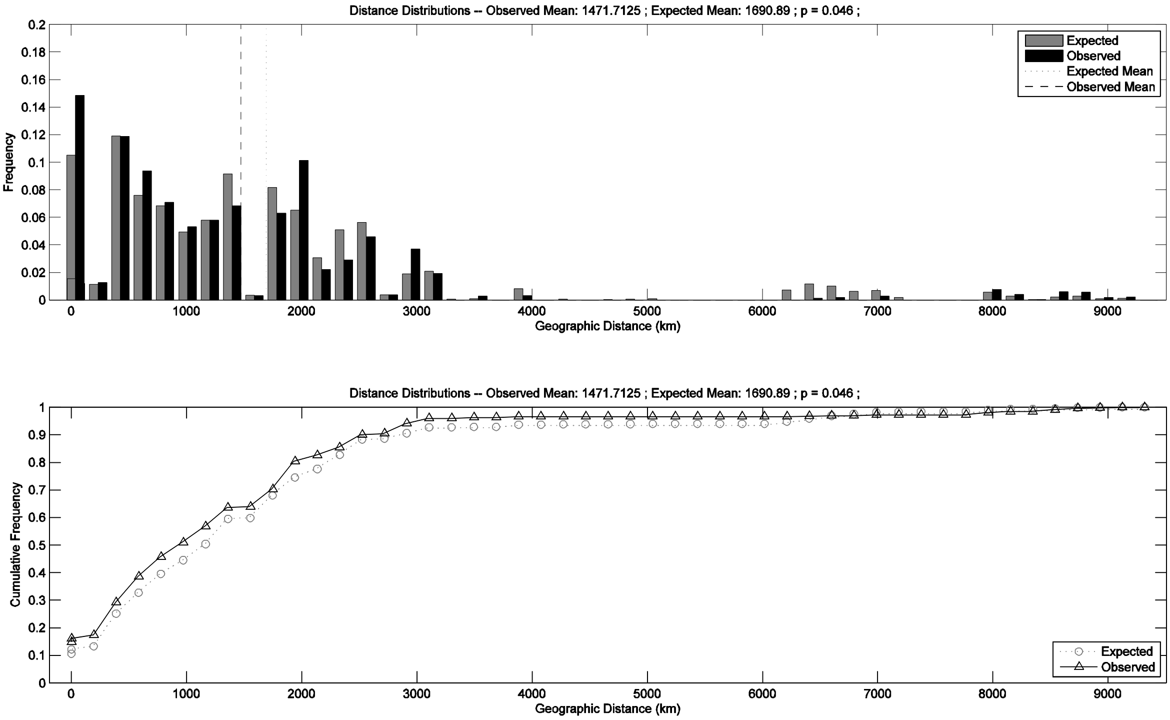

b) Haplotype-by-haplotype analysis of the complete C.ciconia dataset. The number of occurances of each haplotype (n) and significance level (p) are given along with the haplotypes specific SAShA statistics. The expected distribution is represented with circles and the observed by triangles.

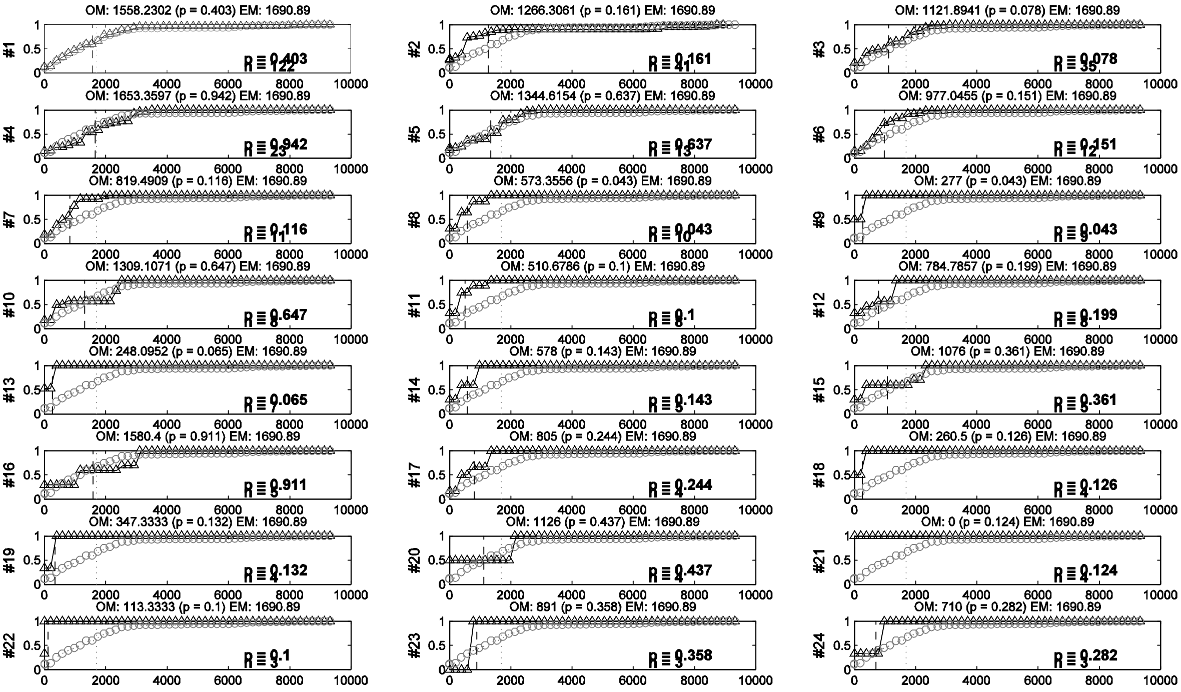

Fig. S2 Observed versus expected mean distribution of alleles with the most common haplotype (CC003) removed showing that the global distribution of CC003 overshadows some degree of underlying population structure.

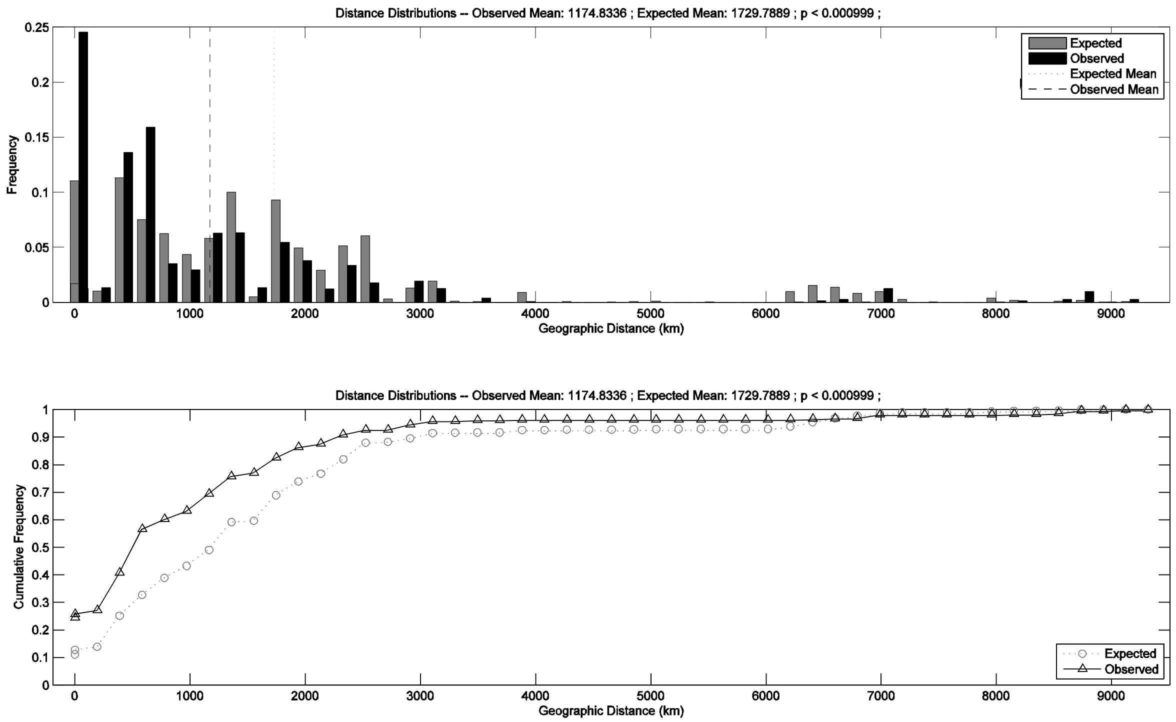

Supplement: Supplementary file 2 [file ece30003-4881-SD2.pdf]
